# Integrated enzymatic and sonication strategy for sustainable soybean processing: from cell wall deconstruction to product separation

**DOI:** 10.1186/s40643-025-00991-5

**Published:** 2026-01-27

**Authors:** Afranul Qader Ovi, Lu-Kwang Ju

**Affiliations:** 1 Department of Chemical, Biomolecular, and Corrosion Engineering, The University of Akron, Akron, OH USA; 2 Present Address: Agricen Sciences, Pilot Point, TX USA

**Keywords:** Enzymatic soybean processing, Solid-state fermentation, Cell wall degradation, Oil body recovery, Carbohydrate monomerization, Native protein preservation

## Abstract

Conventional soybean processing relies on hexane extraction and high-temperature treatments, which cause protein denaturation and hinder the separate recovery of oil, protein, and carbohydrate. To address this limitation, this study aimed to establish a sustainable enzymatic soybean processing (ESP) strategy for separate collection of all three major components: intact oil bodies, undenatured protein, and monomerized carbohydrate. This is the first demonstration that ESP efficiency can be significantly enhanced by integrating pulsed sonication. Multi-enzyme systems were produced via solid-state fermentation (SSF) of soyhull by *Aspergillus niger* and applied to cracked soybean particles. Screening of 15 SSF enzyme extracts revealed that pectinase, polygalacturonase, and invertase were the limiting carbohydrase activities for cell wall degradation. Effects of processing variables including protease activity, reaction media, and reaction time were evaluated to minimize protein loss. Using water instead of citrate buffer as reaction medium and limiting processing time to ≤ 48 h reduced protein dissolution to below 20%. ESP was further enhanced through pulsed probe-sonication (12 W/mL, 1 s on/23 s off), which reduced processing time to 24 h while increasing carbohydrate solubilization to up to approximately 90% depending on enzyme loading. Simple centrifugation enabled efficient fractionation into intact oil bodies (100%), native proteins (≈70%), and hydrolysates containing soluble proteins (≈30%) and monomerized carbohydrate (≈90%). These findings demonstrate an integrated enzymatic-sonication approach that enables hexane-free, low-temperature soybean processing with minimally denatured, high-value products and offers a pathway for sustainable soybean biorefinery.

**Graphical abstract:**

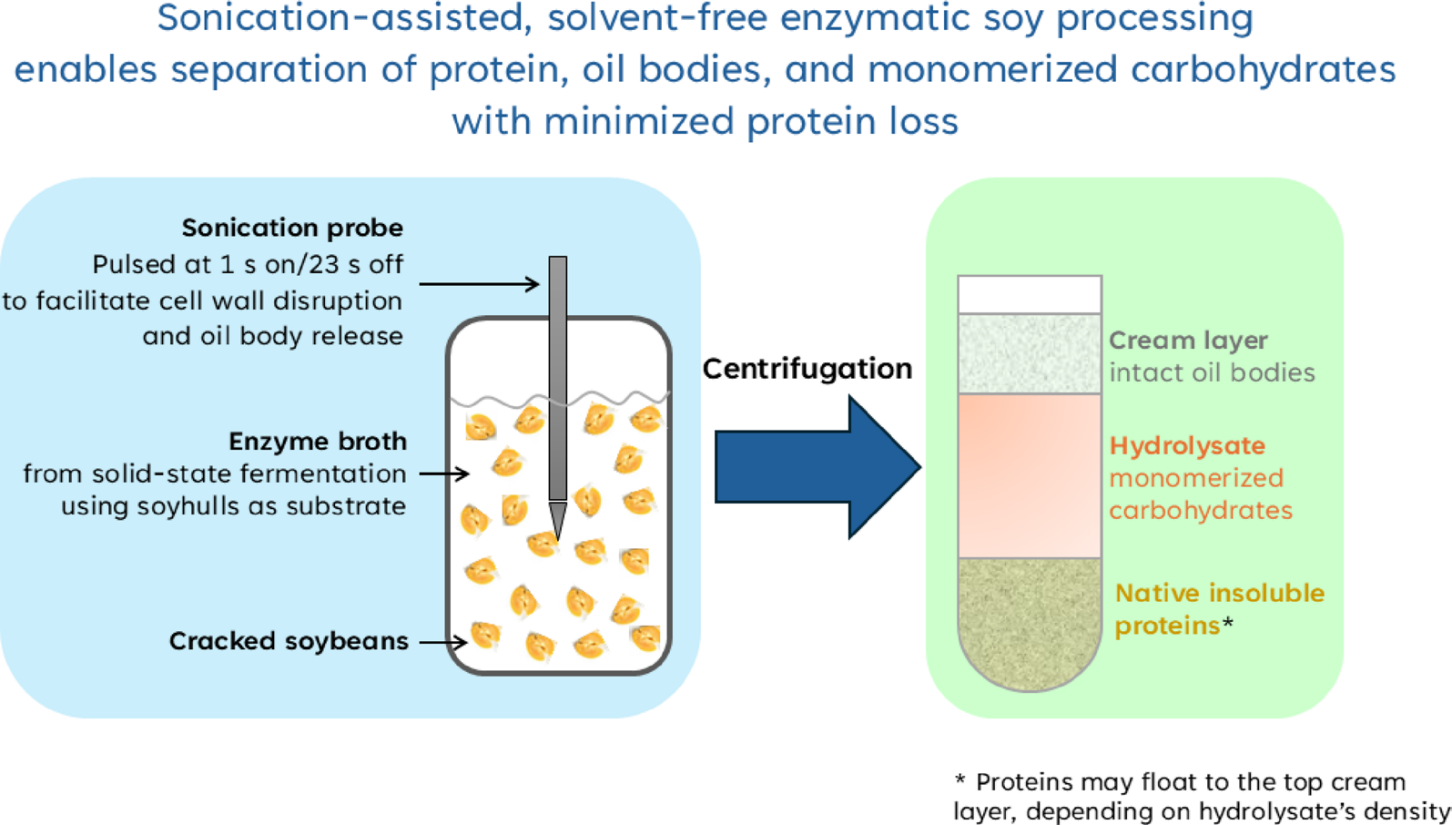

**Supplementary Information:**

The online version contains supplementary material available at 10.1186/s40643-025-00991-5.

## Introduction

Cultivated for centuries in eastern Asia, soy is adaptable to diverse soil and climate conditions, making it a versatile and widely grown crop for food, feed, and industrial uses. Chemically, soybeans are composed of three main groups: approximately 18–20% oil, 40% protein, and 25–30% carbohydrate (Medic et al. 2014). The historical evolution of soybean applications in different regions has led to the development of processing methods that focus on extracting specific components, such as oil or protein (Li et al. 2012; Loman and Ju 2016). Soybean oil is valuable for human consumption and, more recently, for the production of biofuel and biobased products (Loman and Ju 2016), while soybean protein offers a beneficial amino acid profile for both human and animal diets (Gatlin et al. 2007). Different processing methods leave the remaining components in different chemical and physical forms. These residual materials contain high levels of biodegradable organics. If they are not recovered, they must be treated as waste, which incurs significant disposal costs. Due to their varying properties (Bainy et al. 2008; Loman and Ju 2017), the byproducts or wastes from different processes can require distinct and complex methods for subsequent processing, which can be both challenging and costly to customize (Mateos-Aparicio et al. 2010; Karp et al. 2011; Pierce et al. 2016). Therefore, developing a simplified, unified soybean processing method that effectively recovers and separates all major components can provide substantial benefits in cost, sustainability, and resource optimization.

Currently, large-scale soybean processing commonly starts with steps for maximizing oil collection, such as hexane extraction. Despite its efficiency, hexane poses environmental and safety concerns due to its flammability and reliance on petroleum-derived resources (Loman et al. 2018a). These concerns have led to a growing interest in hexane-free, enzyme-assisted aqueous processes to collect oil from soybeans (Jung et al. 2009; Campbell et al. 2011) and other oilseeds (Long et al. 2011; Mat Yusoff et al. 2015; Mechqoq et al. 2021; Dias et al. 2022; Tamborrino et al. 2023; Zhang et al. 2024a; Sorita et al. 2024; Wang et al. 2025; Domínguez-Pérez et al. 2025). For soybean oil collection, these enzymatic methods often use full-fat soy flakes or flour as starting materials (Lamsal et al. 2006; Jung and Mahfuz 2009). Oil collection efficiencies of up to 80% from full-fat soybean meal or flakes have been achieved by using cellulase and protease combinations to enhance oil release by breaking down the entrapping biomass (Lamsal et al. 2006; Campbell et al. 2011). Beyond cellulase and protease, recent studies have often evaluated various combinations of pectinase (including pectinmethylesterase and polygalacturonase) and hemicellulase (including xylanase) for enhancing oil recovery from oil seeds (Passos et al. 2009; Mechqoq et al. 2021; Tamborrino et al. 2023; Zhang et al. 2024a; Sorita et al. 2024; Wang et al. 2025; Domínguez-Pérez et al. 2025). Further coupling the enzymatic treatment with mechanical methods such as sonication and extrusion has demonstrated synergistic effects in disrupting biomass and improving oil release (Kapchie et al. 2008; Zhang et al. 2024a; Wang et al. 2025). However, the use of protease in many earlier studies is associated with protein degradation, which can impact the economic viability particularly for soybeans whose high protein content is an important part of its overall value (Loman et al. 2018b).

Structurally, soybeans consist of an outer hull (comprising approximately 8% of bean weight), the plumule and hypocotyl (about 2%), and two cotyledons (about 90%) (Medic et al. 2014). Cotyledons are made up of cylindrical cells (30 μm × 70 μm) containing oil bodies and protein bodies, enclosed by a protective cell wall of polymeric structural carbohydrates, primarily pectin, hemicellulose, and cellulose (Loman et al. 2018b; Kohli and Singha 2024). Protein bodies are nearly spherical, with diameters of 2–10 μm. Oil bodies (also known as oleosomes and spherosomes) are much smaller, about 0.2–0.5 μm in diameter, filling the interstitial space between protein bodies. Oil bodies are stabilized by a phospholipid layer interspersed with amphipathic proteins known as oleosins (Iwanaga et al. 2008).

In the commonly used processing with hexane extraction, soybeans go through treatments such as extrusion, heating, grinding, and pressing. The soybean flakes and flour used as the starting material in prior enzyme-assisted aqueous extraction studies had also been exposed to many of these thermal and mechanical stresses (Lamsal et al. 2006; Jung and Mahfuz 2009). These treatments disrupt cellular structures as well as the integrity of subcellular protein and oil bodies. After hexane extraction, the remaining meal is a complex mixture of mainly protein and carbohydrate, which have been mechanically mixed and pressed together and become more difficult to separate. This also limits the production of protein-enriched products like soy protein concentrate and soy protein isolate. Currently, soybean meal is primarily used in animal and aquaculture feed, where higher protein content is not essential (Ogunkoya et al. 2006; Cai et al. 2024). Besides increasing the protein content, it is desirable to separate carbohydrates from protein. Soybean carbohydrates are predominantly indigestible galacto-oligosaccharides like raffinose and stachyose, along with insoluble polysaccharides including pectin, hemicellulose, and cellulose. Their presence reduces the digestibility of soybean products for humans and other monogastric animals and fish, thereby lowering the value in food and feed applications.

Considering the structure of soybean cells and the largely separated presence of structural carbohydrates in cell walls, protein in protein bodies, and oil in oil bodies, Ju and coworkers hypothesized that it is possible to enzymatically break the cell-wall polysaccharides into their constituent monomeric sugars (i.e., carbohydrate monomerization) and release the intact protein bodies and oil bodies for separate collection (Loman et al. 2018b). By controlling the pH of enzymatic processing near the isoelectric point (pI) of main soybean proteins, the protein bodies and intact oil bodies would remain insoluble in the aqueous hydrolysate of carbohydrate and, therefore, could be easily separated. Intact oil bodies have high-value applications in food, cosmetics, and pharmaceuticals where stable oil-in-water systems are required (Iwanaga et al. 2007). These oil bodies offer excellent stability, reducing the need of synthetic surfactants in formulations (Iwanaga et al. 2008). The protein produced by this process is also in its native form, without denaturation by the heat and mechanical stresses involved in the conventional soybean processing. By enzymatic processing, soluble oligosaccharides such as sucrose, raffinose, and stachyose are also monomerized, thereby eliminating the indigestibility concern. Instead of being a low-value byproduct or waste like soybean molasses, the monomerized carbohydrate offers additional energy when used in feed and food and can be used as a ready fermentation feedstock (Loman et al. 2016) for producing biofuels and value-added chemicals (Wang et al. 2008; Mielenz et al. 2009). Overall, this enzymatic soybean processing method can significantly enhance the utility and economic value of soybean products.

Despite these promising advantages and the demonstrated feasibility of enzymatic soybean processing, earlier enzyme-assisted soybean processing studies left three major challenges unresolved: (1) identifying which carbohydrase activities truly limit cell-wall breakdown, (2) minimizing protein loss caused by protease and buffer conditions, and (3) reducing the long processing times that hinder economic feasibility. These limitations have prevented broader industrial adoption. Numerous studies have demonstrated that sonication significantly enhances enzymatic hydrolysis by improving mass transfer, enzyme–substrate affinity, and reaction kinetics (Wang et al. 2017, 2020; Soares et al. 2019). Ultrasound pre-treatment or co-application with enzymes has been shown to accelerate reaction rates (V_max_) while keeping the Michaelis–Menten constant K_m_ unchanged, leading to lower enzyme requirement (Soares et al. 2019). Another group of study on sonication-assisted oil extraction from rapeseed and flaxseed showed that proper ultrasonic treatment enhanced oil extraction and yield (Zhang et al. 2008; Wei et al. 2008). Chandrapala and coworkers studied the effect of ultrasonication on thermal and structural characteristics of protein and reported no significant deformation (Chandrapala et al. 2011). Ju and coworkers demonstrated how pulsed sonication can help separate oil bodies from protein bodies in enzyme assisted soybean processing (Loman et al. 2018b).

This study explicitly addresses these knowledge gaps by: (1) systematically profiling limiting carbohydrase activities in enzyme extracts from *Aspergillus niger* solid-state fermentation (SSF), (2) evaluating strategies to minimize protein dissolution during processing, and (3) for the first time, integrating pulsed sonication with enzymatic treatment to accelerate cell-wall breakdown while preserving component integrity. The combination of these approaches represents a novel integrated strategy that enhances processing efficiency, preserves native protein, and enables sustainable soybean biorefining beyond what has been achieved in prior studies.

## Materials and methods

### Culture, chemicals and equipment

The filamentous fungus *Aspergillus niger* NRRL 322, obtained from the USDA ARS Culture Collection, was used for enzyme production. Potato Dextrose Broth (PDB) and Potato Dextrose Agar (PDA), used for culture propagation, maintenance and spore production, were both sourced from BD Difco (Sparks, MD, USA). A 1% (w/v) solution of Tween 80 (Sigma-Aldrich, St. Louis, MO, USA) was used for spore collection.

Two primary agricultural substrates were used: soyhulls for solid-state fermentation (SSF) and full-fatted, dehulled, cracked soybeans for enzymatic soybean processing (ESP). Both were provided by Archer Daniels Midland Company (ADM; Decatur, IL, USA). Prior to use, cracked soybeans were ground and sieved to 0.85–2 mm. All chemicals used in SSF nutrient media were of analytical grade and purchased from Sigma-Aldrich. Polygalacturonic acid (PGA; ≥ 95% purity, MW 25,000–50,000 Da; Product No. 81325, Sigma-Aldrich) was used as substrate in polygalacturonase activity assays. Citrate buffers (0.05–2 M, pH 4.8) were prepared from citric acid and sodium citrate (Sigma-Aldrich). Sodium azide (NaN_3_, 99% purity; Sigma-Aldrich) was added to inhibit microbial contamination during ESP experiments.

For carbohydrate analysis, a High-Performance Liquid Chromatography (HPLC) system (Agilent 1100 Series, Agilent Technologies, Santa Clara, CA, USA) was used, equipped with a SUPELCOGEL Pb column (30 cm × 7.8 mm) and guard column (5 cm × 4.6 mm), both from Sigma-Aldrich. Samples were filtered using 0.22 µm polyvinylidene difluoride (PVDF) syringe filters (Millipore, Burlington, MA, USA). For protein concentration determination using the Total Kjeldahl Nitrogen (TKN) method, the sample digestion was performed using selenium catalyst mixtures and Kjeldahl digestion apparatus (Labconco Corporation, Kansas City, MO, USA), and the subsequent distillation was done using a Labconco RapidStill II distillation unit. Protease activity was measured using a Fluorescent Protease Assay Kit (Pierce/Thermo Scientific, Catalog No. 23266, Waltham, MA, USA) and analyzed using a microplate reader (Infinite 200 PRO, Tecan Group Ltd., Männedorf, Switzerland). Sonication was applied using a probe sonicator (Qsonica Q700, Newtown, CT, USA) or a sonication bath (Branson 3800, Emerson, Brookfield, CT, USA). Absorbance was measured using a spectrophotometer (UV-1601, Shimadzu; Columbia, MD). Enzymatic soybean processing experiments were conducted in a shaker (Thermo Scientific MaxQ 5000 Incubating/Refrigerating floor shaker; Ashville, NC). Three centrifuges were used in this study: a basket-type centrifugal filtration machine (IEC; Chattanooga, TN), a Sorvall Legend X1R centrifuge (Thermo Scientific; Waltham, MA), and a Sorvall RC 5C centrifuge (DuPont; Wilmington, DE).

### Culture preparation

*A. niger* (NRRL 322) was acquired as spores from the USDA Agricultural Research Service Culture Collection and maintained at − 80 °C. For culture preparation, thawed spores were transferred with a sterile loop into 100 mL PDB (20 g/L) in a 250 mL flask and incubated at 32 °C, 200 rpm for 48–72 h until pellet growth plateaued. The resulting culture was either stored in 20% glycerol at − 80 °C or used for spore production. For spore production, 1 mL culture was spread on 20 g/L PDA plates and incubated at 30 °C, > 70% relative humidity (RH) until sporulation. Spores were harvested by adding 20 mL of 1% Tween 80 and gently dislodging with a sterile loop. The collected spore suspension in Tween 80 was stored at 4 °C and used for SSF inoculation within one week.

### Enzyme production

Enzymes were produced by *A. niger* SSF using soyhull as the primary carbon (C) source and the inducer for carbohydrase genes. Large soyhull particles (> 3.5 mm) were used to minimize clustering and enhance bed porosity for better oxygen and heat transfer. SSF was conducted in 250 mL beakers, each containing 10 g soyhull and 15 mL aqueous nutrient medium (i.e., ~ 60% water content). The nutrient medium included variable nitrogen sources (ammonium sulfate, ammonium acetate, and urea) alongside 6.34 g/L KH_2_PO_4_, 3.70 g/L MgSO_4_·7H_2_O, 1.32 g/L CaCl_2_·2H_2_O, 0.2 mL/L Tween 80, and 1 mL/L of trace element solution (5 mg/L FeSO_4_·7H_2_O, 1.6 mg/L MnSO_4_·4H_2_O, 2 mg/L CoCl_2_·2H_2_O, 1.4 mg/L ZnSO_4_·7H_2_O). The mixture was autoclaved (121 °C, 15 min), cooled, and then inoculated with 1 mL of *A. niger* spore suspension or 1 mL PDB grown culture. Fifteen distinct SSF systems were cultivated at 21.0–24.5 °C and 16–23% RH but with several other varying parameters, as detailed below and summarized in Table 1.

**Table 1:** N source composition, inoculum source, harvesting time, and initial medium pH used in different SSF systems, to generate different enzyme activity profiles (Table 2) for the limiting carbohydrase determination study (Sect. ”Determination of limiting carbohydrase activities in SSF enzyme extracts for ESP”)

SSF systems	N source (%)			Inoculum source	Harvesting time (h)	Initial medium pH
	Ammonium sulfate	Ammonium acetate	Urea			
1	82	0	18	PDA spores	120	6.0
2	82	0	18	PDA spores	72	6.0
3	82	0	18	PDA spores	120	5.0
4	82	0	18	PDA spores	72	5.0
5	82	0	18	PDA spores	110	7.0
6*	82	0	18	PDA spores	120	5.5
7	0	82	18	PDA spores	120	3.5
8	20	0	80	SSF spores	216	3.5
9	20	0	80	SSF spores	216	9.0
10	100	0	0	SSF spores	216	4.5
11	82	0	18	PDB cells	312	6.0
12**	82	0	18	PDA spores	312	6.0
13	82	0	18	PDA spores	144	6.0
14	82	0	18	SSF spores	144	6.0
15	200	0	0	SSF spores	144	3.5

^*^Supplemented with 5 g additional soymeal

^**^Grown at higher than 90% RH

For nitrogen (N) source composition, nine systems used ammonium sulfate and urea (commonly at a 82:18 ratio, except for the 20:80 ratio used in Systems 8 and 9), while others included 100% ammonium sulfate (System 10), 200% ammonium sulfate (System 15), or substituted ammonium sulfate by ammonium acetate (System 7). Note that 100% ammonium sulfate was 34.4 g/L, 100% ammonium acetate was 40.1 g/L, and 100% urea was 15.7 g/L, each corresponding to 7.2 g/L N in the nutrient medium. For inoculum source, most systems used PDA spores; however, Systems 8–10 and 14–15 used spores harvested from SSF System 1 (with 100 mL 1% Tween 80 per beaker, shaken at 200 rpm for 2 h), while System 11 was inoculated with 1 mL PDB grown cells. Fermentation duration ranged from 72 h (Systems 2 and 4) to 312 h (Systems 11 and 12). Initial nutrient medium pH, adjusted before dosing to soyhull, ranged from 3.5 (Systems 7, 8 and 15) to 9 (System 9). There were some additional modifications: System 6 was supplemented with 5 g additional soymeal to increase carbon and nitrogen loading, and System 12 was cultured at a relative humidity above 90%. These factors all affected cell growth, enzyme production, and/or the activity profile of harvested enzyme extract (as given later in Table 2 in the Results and Discussion section).

**Table 2:** Carbohydrases loadings and resultant % volume reductions after 72 h ESP made for determining the limiting carbohydrases in SSF enzyme extracts

Enzyme extract	Enzyme loading, FPU or U/g soybean									% Volume reduction
	Invertase	α- Galactosidase	Pectinase	Poly-galacturonase	Xylanase	Cellulase	Endo-glucanase	Exo-glucanase	β-Glucosidase	
1	43.5 ± 1.3	135.7 ± 4.1	66.9 ± 2.0	12.4 ± 0.4	403 ± 4	5.4 ± 0.2	47.0 ± 1.4	0.6 ± 0.1	1.1 ± 0.1	86.5 ± 0.7
2	29.6 ± 1.2	88.2 ± 3.7	72.1 ± 3.0	11.4 ± 0.5	674 ± 8	5.7 ± 0.2	35.4 ± 1.5	2.9 ± 0.1	2.1 ± 0.5	72.5 ± 0.8
3	33.6 ± 0.9	84.9 ± 2.4	63.3 ± 1.8	11.4 ± 0.3	589 ± 18	4.1 ± 0.1	19.8 ± 0.5	2.0 ± 0.1	4.7 ± 0.1	70.0 ± 1.5
4	38.1 ± 1.1	114.8 ± 3.4	64.8 ± 1.9	10.5 ± 0.3	611 ± 12	3.8 ± 0.4	25.6 ± 0.8	1.1 ± 0.3	4.1 ± 0.1	70.1 ± 1.3
5	32.0 ± 1.3	49.5 ± 2.1	57.5 ± 2.4	11.3 ± 0.5	574 ± 11	4.0 ± 0.2	20.6 ± 0.9	3.6 ± 0.2	10.4 ± 0.4	63.2 ± 1.6
6	13.5 ± 0.4	19.5 ± 0.5	26.9 ± 0.7	12.0 ± 0.3	261 ± 6	1.4 ± 0.1	19.4 ± 0.5	1.3 ± 0.4	9.8 ± 0.3	38.2 ± 1.3
7	12.0 ± 0.5	26.0 ± 1.1	27.1 ± 1.1	11.2 ± 0.5	256 ± 7	2.6 ± 0.7	17.4 ± 0.7	1.2 ± 0.1	9.3 ± 0.4	32.1 ± 0.3
8	36.1 ± 1.0	85.5 ± 2.4	74.2 ± 2.1	9.1 ± 0.3	502 ± 14	3.1 ± 0.1	24.4 ± 0.7	0.1 ± 0.2	6.5 ± 0.2	73.6 ± 2.5
9	39.9 ± 1.2	229.4 ± 6.9	29.1 ± 0.9	1.9 ± 0.1	299 ± 9	4.6 ± 0.3	25.0 ± 0.7	1.1 ± 0.5	6.1 ± 0.2	35.2 ± 1.6
10	18.5 ± 0.6	177.5 ± 5.3	87.2 ± 2.6	10.9 ± 0.3	499 ± 15	4.2 ± 0.4	28.3 ± 0.8	3.3 ± 0.1	3.6 ± 0.1	81.9 ± 1.0
11	38.1 ± 1.6	68.2 ± 2.9	67.4 ± 2.8	12.8 ± 0.5	733 ± 8	3.5 ± 0.1	23.2 ± 1.0	2.7 ± 0.3	0.7 ± 0.2	72.4 ± 0.6
12	47.9 ± 1.3	81.9 ± 2.3	48.6 ± 1.3	9.7 ± 0.3	418 ± 12	3.0 ± 0.1	24.2 ± 0.7	3.2 ± 0.1	0.5 ± 0.1	54.2 ± 6.4
13	29.0 ± 1.2	96.6 ± 4.1	67.1 ± 2.8	7.4 ± 0.3	626 ± 26	3.1 ± 0.6	23.8 ± 1.0	1.9 ± 0.1	7.4 ± 0.3	60.6 ± 7.1
14	57.8 ± 2.4	147.2 ± 6.2	54.0 ± 2.3	4.5 ± 0.2	233 ± 10	3.4 ± 0.1	23.9 ± 1.0	4.2 ± 0.2	1.6 ± 0.1	66.6 ± 3.4
15	30.4 ± 0.8	107.9 ± 3.0	80.1 ± 2.2	9.9 ± 0.3	433 ± 12	3.7 ± 0.5	21.9 ± 0.6	1.0 ± 0.6	3.7 ± 0.1	69.3 ± 0.4

ESP experiments made with 6.65 mL enzyme extract and 3.35 mL 0.5 M citrate buffer (pH 4.8) at 45 °C and 250 rpm orbital shaking

Standard deviations for enzyme loadings were from triplicate activity measurements, and those for % volume reduction were from duplicate ESP systems for each enzyme extract

After fermentation, 20 mL deionized water was added to the SSF mixture and stirred, to allow measurement of harvesting pH. Next, 40 mL of 0.1 M citrate buffer (pH 4.8) was added, followed by 2 h shaking at 200 rpm and 32 °C to extract enzymes into the buffer. The mixture was then filtered through the rotating bucket centrifuge using a mesh of 0.25 mm^2^ opening to remove large solids, and the filtrate was centrifuged again at 4648 × g for 25 min to precipitate the fine solids. The supernatant was collected and frozen at − 20 °C for later use as the enzyme extract to process cracked soybean particles. For activity analysis, three aliquots were taken from each enzyme extract, and multiple enzyme activities were assayed, with mean values and standard deviations reported in this work.

The variations in N source type and concentration, inoculum, processing time, and environmental condition were purposefully designed to generate enzyme extracts with distinct carbohydrase profiles, enabling the subsequent identification of limiting enzyme activities during enzymatic soybean processing. All other studies were conducted using the extracts produced according to SSF System 1. The SSF enzyme extract 1 was measured to contain 23.3 ± 0.7 g/L proteins and residual soluble carbohydrates: 6.05 ± 0.12 g/L glucose, 0.83 ± 0.13 g/L xylose, 0.49 ± 0.06 g/L galactose, 0.23 ± 0.12 g/L arabinose, and 0.48 ± 0.10 g/L fructose. Stachyose, raffinose, sucrose, and melibiose were undetectable. These background concentrations were subtracted from the measured protein and carbohydrate concentrations in ESP experiments to estimate the protein and carbohydrate releases attributable to soybean particles.

### Protein dissolution and carbohydrate release and monomerization from soybean particles

To establish baseline profiles for evaluating ESP, a set of control experiments were conducted to assess the release and solubilization of carbohydrates and proteins from soybean particles in the absence of SSF enzyme extract. In these experiments, similar to the later described ESP experiments, 1 g of ground soybean particles was added to 10 mL of deionized water along with 0.1 mL of a 50 g/L sodium azide (NaN_3_) solution in 20 mL glass vials. NaN_3_ was included to inhibit microbial growth during incubation. Soybeans naturally contain endogenous carbohydrases and proteases that facilitate nutrient mobilization during germination (Han et al. 2013; Hu et al. 2021; Gunathunga et al. 2024). To investigate the influence of temperature on the activity of these native enzymes and the resulting protein dissolution and carbohydrate solubilization and monomerization, experiments were carried out at four temperatures: 2 °C, 20 °C, 25 °C, and 45 °C. Each condition was tested in triplicate. Following a 96-h incubation at the designated temperatures, the contents of each vial were transferred into 15 mL centrifuge tubes and centrifuged at 4648 × g for 25 min to separate supernatants from solids. The resulting supernatants were then analyzed for carbohydrate and protein concentrations and used to determine % carbohydrate solubilization, % carbohydrate monomerization, and % protein dissolution, as described in detail in the Analytical Methods section.

### Enzymatic soybean processing experiments

With more details given later, the enzymatic soybean processing (ESP) experiments done in this study differed in the enzymes used (produced under different SSF conditions as described earlier in Sect. ”Enzyme production”); enzyme loading: 1X, 5X, or 7.5X (1X corresponding to 1.33 mL SSF enzyme extract per 1 g soybean particles); liquid volume (including enzyme extract and dilution medium): 5 or 10 mL; dilution medium: deionized water or pH 4.8 citrate buffer at 0.05 M, 0.1 M, 0.5 M, 1 M, or 2 M concentration; processing time: 24–168 h; processing temperature: 2, 20, 25, 45, or 50 °C; processing method: without sonication, with pulsed sonication by a sonication probe, or with intermittent sonication in a sonication bath; and/or shaking and mixing condition: orbital shaking, magnetic stirring, or no mixing. All ESP experiments were made with 1 g soybean particles in a 20 mL glass vial. For controlling microbial growth, a 50 g/L NaN_3_ solution was added at the ratio of 0.1 mL per 10 mL liquid volume. Accordingly, for example, a system of 1X enzyme loading and 10 mL liquid volume would have 1 g soybean particles, 1.33 mL enzyme extract, 8.67 mL reaction medium, and 0.1 mL NaN_3_ solution; and a system of 1X enzyme loading and 5 mL liquid volume would have 1 g soybean particles, 1.33 mL enzyme extract, 3.67 mL reaction medium, and 0.05 mL NaN_3_ solution; and a system of 7.5X enzyme loading and 10 mL liquid volume contained 1 g soybean particles, 10 mL enzyme extract, and 0.1 mL NaN_3_ solution. For non-sonicated systems, the vials were horizontally attached to the orbital shaker operating at 250 rpm for the studied processing time. For bath-sonicated systems, the vials were horizontally placed in the sonication bath filled with 3.78 L deionized water and sonicated in cycles of 10-min on and 60-min off, with sonication power (while on) of 0.15 W per mL of bath volume. For probe-sonicated systems, the reaction mixture was mixed by a magnetic stirring bar while being pulse-sonicated by a probe in cycles of 1-s on and 23-s off with sonication power (while on) of 12 W per mL of reaction mixture. The probe-sonicated systems were processed for 24 h, with a cumulative sonication (on) time of 1 h. Details of the specific ESP experiments done for different study purposes are given in the following subsections.

#### Determination of limiting carbohydrase activities in SSF enzyme extracts for ESP

15 enzyme extracts obtained from different SSF systems (Table 1) were used in this study. The ESP experiments using these enzyme extracts were conducted in duplicate, with 5X enzyme loading in 10 mL of 0.5 M citrate buffer (pH 4.8) as the dilution medium. The carbohydrases loadings used in these experiments are given in Table 1, with the activities measured by using the procedures described in Sect. “Sonication-facilitated enzymatic soybean processing (ESP)”. The mixture was allowed to react for 72 h in an orbital shaker at 45 °C and 250 rpm. Then, the processed mixture was transferred to a 15 mL centrifuge tube and centrifuged at 4648 g for 25 min. This centrifugation packed the remaining, not fully broken, soybean particles in the bottom of the centrifugation tube. The height of this packed bed was measured and used to determine the % particle volume reduction, which was used as a convenient and effective index for screening the extent of completion of ESP (see Sect. 2.5.5 for more details). Analysis of variance (ANOVA) was done to evaluate the correlation of measured % particle volume reduction with all carbohydrase activities involved. Presumably, the carbohydrases with large p values were present in excess, so that the SSF enzyme extracts with different activities of these carbohydrases did not significantly affect the % particle volume reduction. On the other hand, the carbohydrases with low *p* values (< 0.05) were limiting for ESP. After the limiting carbohydrases were identified, a correlation equation was established between the measured % particle volume reduction and the limiting activities.

#### Effects of processing factors on protein loss

Here, protein loss is defined as the percentage of N-containing (presumably proteinaceous) materials that migrated from soybean particles to the aqueous medium during soybean processing. This migration may result from simple dissolution of native proteins or be facilitated by enzymatic breakdown of the soybean matrix. To quantify this, total N content was measured in both the original soybean particles and the liquid hydrolysate using the Kjeldahl method. The initial N content of soybean particles was found to range from 6.4 to 6.9% on a dry weight basis. Following soybean processing, the N content in the aqueous phase was measured and corrected for any N contribution from the enzyme extract itself, which varied across different extracts from 0.03% to 0.08%. The percentage N migration (protein loss) was then calculated by comparing the corrected N in aqueous medium to the total initial N in soybean particles. The processing factors assessed, along with the experiments conducted for their evaluation, are detailed in the following subsections.

##### Effect of protease

20–30% of the proteins in soybean cotyledons are water soluble (Zhang et al. 2024b). The remaining storage proteins are insoluble near their isoelectric point pI ≈ 4.5 (Guo 2009), but protease can break them into smaller soluble peptides (Islam et al. 2020). To investigate the effect of protease on dissolution of these insoluble soy proteins, experiments were done with a laboratory grade soy protein isolate (SPI, MP Biomedicals, Solon, OH) having 90.8% protein, 4% ash, 4.4% moisture, and 0.4% fat. Three systems were designed, each consisting of triplicate 20-mL glass vials. Each of the nine vials received 0.474 g SPI (providing 0.43 g protein, equivalent to the 43% protein content in 1 g soybean particles), 0.1 mL of 50 g/L NaN_3_ (for microbial control), and 8.67 mL of 0.5 M citrate buffer (pH 4.8, serving as the dilution medium). The systems differed in the additional 1.33 mL of aqueous medium added to each vial. In one system, the additional medium was an SSF enzyme extract containing 37.4 ± 2.4 BAEE U/mL protease and various carbohydrases: cellulase, 0.84 ± 0.04 FPU/mL; pectinase, 8.7 ± 0.2 U/mL; α-galactosidase, 17.2 ± 0.7 U/mL; invertase, 6.3 ± 0.2 U/mL; and xylanase, 99.4 ± 10.0 U/mL. (BAEE stands for N-benzoyl-L-arginine ethyl ester, the substrate used in the protease assay. FPU stands for Filter Paper Unit.) In the other two systems, the media were deactivated enzyme extract (by heating at 95 °C for 60 min) and deionized water, respectively. The rationale for this design was to distinguish the impact of active protease from other potential contributors to protein dissolution. The deactivated enzyme extract accounts for the effects of extract composition (e.g., N content, ionic strength, non-enzymatic solubilization), while the deionized water group establishes a baseline for passive solubilization.

All vials were held horizontally and mixed for 72 h in an orbital shaker operating at 250 rpm and 50 °C, before the contents were transferred into tubes and centrifuged at 4648 g for 25 min to settle insoluble proteins. 1 mL supernatant was then collected from each vial and measured for protein by the TKN method, for determination of the % protein dissolution.

##### Effect of processing time

Two sets of experiments were carried out to investigate the trends of protein dissolution over time in both enzyme-free control and 1X enzymatic systems. Each group consisted of 14 sacrificial systems, incubated at 45 °C with orbital shaking at 250 rpm (horizontally mounted). Every 24 h, two systems (duplicates) were collected, centrifuged for 25 min at 4648 g and measured for % particle volume reduction. All systems contained 1 g soybean particles in a total liquid volume of 10 mL, using 0.5 M citrate buffer (pH 4.8) as the dilution medium. In enzymatic systems, 1.33 mL of the 10 mL was SSF enzyme extract (System 1 in Table 1), while the remaining 8.67 mL was buffer. In the enzyme-free controls, the full 10 mL consisted of buffer only. The enzyme extract contained the following activities per mL: 0.84 ± 0.04 FPU cellulase, 8.7 ± 0.2 U pectinase, 17.2 ± 0.7 U α-galactosidase, 6.3 ± 0.2 U invertase, 99 ± 10 U xylanase, and 37 ± 2 BAEE U protease.

##### Effect of dilution medium

To evaluate the effect of citrate buffer concentration used as the dilution medium, we designed 12 control systems using 10 mL medium with varying buffer molarities and operating conditions. The systems were operated for 96 h at 45 °C, with two sets of duplicate samples: one under orbital shaking (250 rpm) and the other with no shaking. The six buffer strengths compared were: 0 M (deionized water), 0.05 M, 0.1 M, 0.5 M, 1 M, and 2 M citrate buffer, all adjusted to pH 4.8. These variations allowed us to assess the influence of buffer strength and mixing conditions on the dissolution behavior of proteins and carbohydrates during processing.

#### Sonication-facilitated enzymatic soybean processing (ESP)

Two batches of experiments were conducted to investigate whether sonication could enhance enzymatic soybean processing (ESP). In all systems, the enzyme extract was produced by SSF System 1 (Table 1), and deionized water was used as the dilution medium. The first batch explored the effects of different sonication methods—no sonication, bath sonication, and probe sonication—as previously described in Sect. “Enzymatic soybean processing experiments”. Each method was applied to systems containing 1X enzyme loading with either 5 mL or 10 mL total liquid volume. No-sonication and bath-sonication systems were processed for 96 h, while probe-sonicated systems were processed for only 24 h due to the higher intensity of treatment. An additional probe-sonication system containing 10 mL of undiluted enzyme extract (approximately 7.5X loading) was included to examine conditions leading to near-complete (~ 100%) cell wall disruption. The no-sonication systems were incubated at 25 °C with 250 rpm shaking. The bath sonication systems were operated in 10-min on/60-min off cycles at 0.15 W per mL of bath volume. The probe sonication systems were pulse-sonicated in 1-s on/23-s off cycles at 12 W per mL of reaction mixture. Both bath- and probe-sonicated systems were initiated at room temperature (21–24 °C), but actual processing temperatures rose due to sonication: bath-sonicated systems reached 25–28 °C, and probe-sonicated systems 25–29 °C. The on–off sonication cycles were chosen from preliminary tests to prevent excessive heating and minimize enzyme deactivation. After processing, all samples were centrifuged, and three supernatant aliquots (liquid hydrolysates) were collected for protein and carbohydrate analysis.

The second batch of experiments focused on evaluating potential enzyme deactivation caused by pulsed probe sonication. Two enzyme solutions (10 mL each) were subjected to sonication at two power levels, 12 and 21 W/mL, under identical pulsed conditions (1 s on, 23 s off) for 24 h. Enzyme activities (cellulase, pectinase, xylanase, invertase, and α-galactosidase) were measured in triplicate before and after sonication to evaluate potential activity loss caused by the treatment.

### Analytical methods

#### Carbohydrate analysis

Stachyose, raffinose, sucrose, melibiose, glucose, xylose, galactose, arabinose and fructose were quantified by High-Performance Liquid Chromatography (HPLC, LC1100, Agilent Technologies) using a refractive index detector and a SUPELCOGEL-Pb column (30 cm × 7.8 mm) with guard column (5 cm × 4.6 mm, Sigma-Aldrich). The mobile phase was HPLC-grade water (0.5 mL/min), column temperature 70 °C, injection volume 25 µL, and run time 40 min. Samples were filtered through 0.22 µm PVDF syringe filters, and calibration curves were prepared with pure standards. Each sample was analyzed in triplicate. Carbohydrate concentrations in hydrolysates were used to calculate carbohydrate solubilization and monomerization achieved by ESP. Solubilization (%) was defined as the fraction of total soybean carbohydrates (assumed 29% of dry weight (Medic et al. 2014)) detected in hydrolysates. Monomerization (%) was defined as the fraction of soluble carbohydrates present as monomeric sugars (glucose, fructose, galactose, xylose, and arabinose).

#### Protein analysis

The Total Kjeldahl Nitrogen (TKN) method, as described by Kirk (1950), was used to measure the N content in samples, which was then multiplied by 6.25 to estimate the protein content. The detailed procedure is given in Supplementary Materials.

#### Enzyme activities analysis

The activities of α-galactosidase, invertase, pectinase, polygalacturonase (hydrolyzing pectin’s polygalacturonate backbone), xylanase, cellulase and its three main component activities: β-glucosidase, endoglucanase, and exoglucanase, and protease were measured with triplicate samples, and the averages with standard deviations are reported. Cellulase activity is expressed in FPU/mL, protease activity is expressed in BAEE U/mL, and the activities of other enzymes are expressed in U/mL, where 1 U represents the activity that produces 1 μmol target product or hydorlyzes 1 μmol substrate per min. α-Galactosidase activity was measured by the method of Kumar et al. (Kumar et al. 2012), modified by Li et al. (Li et al. 2017). Invertase was assayed using the method of Uma et al. (Uma et al. 2010), as adapted by Li et al. (Li et al. 2017). Pectinase, polygalacturonase, cellulase, and xylanase were determined following Li et al. (Li et al. 2017), Ghose (Ghose 1987), and Bailey et al. (Bailey et al. 1992). β-Glucosidase (cellobiase), endoglucanase, and exoglucanase activities were assayed by the methods of Ju and Afolabi (Ju and Afolabi 1999), modified from Wald et al. (Wald et al. 1984) and Berghem and Petterson (Berghem and Pettersson 1973; Gunjikar et al. 2001). Protease activity was measured with the Pierce Fluorescent Protease Assay Kit (Thermo Scientific, Catalog No. 23266), as described by Li et al. (Li et al. 2017). Detailed procedures are given in Supplementary Materials.

#### Oil analysis

The oil content in the sample was determined by a gravimetric method. A dried and ground sample was accurately weighed (approximately 1 g) and placed in a Soxhlet extractor thimble. The distillation flask was filled with 300 mL of *n*-hexane to extract the oil. The flask was heated using a magnetic stirrer with heating (Fisher Scientific), causing the hexane to vaporize. The vapor was condensed by a condenser mounted above the flask, allowing hexane to drip continuously into the thimble and leach oil from the sample over a 6-h extraction period. After extraction, the oil content was determined based on the weight loss of the dried sample and the weight of the oil collected after solvent removal. A mass balance check was performed, and only results with less than 5% mass balance error were accepted. Accepted values from replicate measurements were averaged to determine the oil content of the sample.

#### Analysis for particle volume reduction by ESP

The reduction of volume of soybean particles achieved by ESP was determined by measuring the height of remaining solids accumulated as a bottom layer in the centrifuge tube after the following centrifugation procedure. 15 mL centrifuge tubes (VWR, Cat No. 21008–089, Radnor, PA) were used for the analysis. The entire ESP mixture was transferred to a tube and centrifuged at 4648 × g for 25 min to separate the solid precipitates from the liquid. The height of precipitates was measured and converted to volume by a correlation (R^2^ = 1) first developed by adding known volumes of water to the centrifuge tube and measuring the corresponding heights. The volume was also determined for the control where the soybean particles were similarly processed (but without shaking or sonication) in enzyme-free dilution medium. The % particle volume reduction due to ESP was calculated using the following equation:

$$\% {\text{particle volume reduction = }}\frac{{{\text{Control volume}} - {\text{ESP volume}}}}{{{\text{Control volume}}}} \times {\mathrm{100}}$$

## Results and discussion

### Carbohydrate release and monomerization and protein dissolution from soybean particles soaked in water

Carbohydrate solubilization from soybean particles soaked in water remained essentially constant (~ 46%) across temperatures from 2 to 45 °C, whereas carbohydrate monomerization increased strongly with temperature, reaching ~ 42% at 45 °C. Protein dissolution peaked near 25 °C, reflecting the temperature sensitivity of soybean protease and its functional role in germination.

**Fig. 1:**
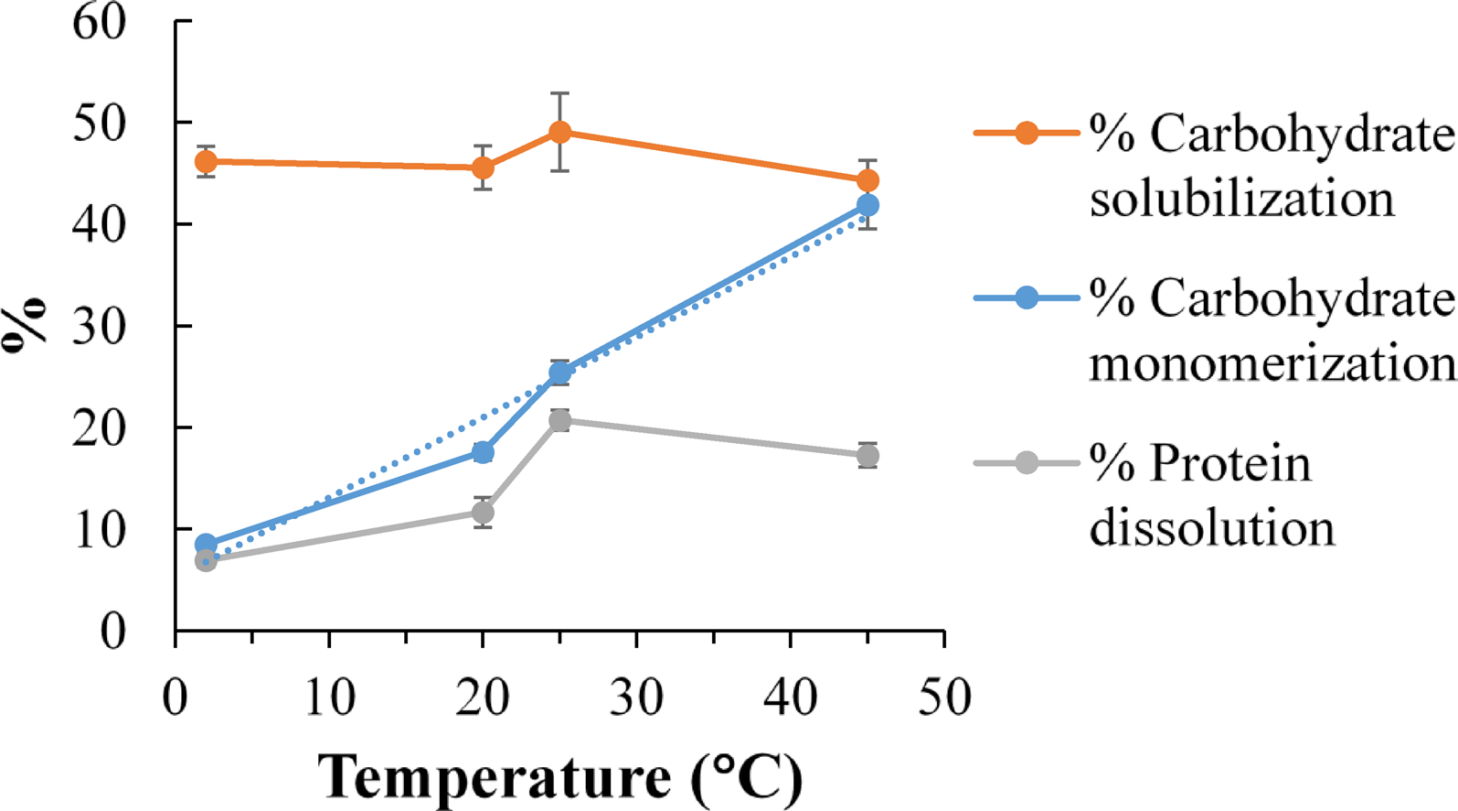
Percentages of carbohydrate solubilization, carbohydrate monomerization, and protein dissolution from soybean particles to deionized water (without SSF enzyme extract) after 96 h of soaking at 2, 20, 25, and 45 °C

Figure 1 shows the profiles of carbohydrate solubilization, carbohydrate monomerization, and protein dissolution at 2, 20, 25, and 45 °C after 96 h soaking in deionized water (without SSF enzyme extract). Carbohydrate solubilization did not vary significantly (*p* > 0.15, paired t-tests), averaging (46.3 ± 1.2)%. By contrast, carbohydrate monomerization increased almost linearly (R^2^ = 0.97) with temperature, from (8.5 ± 0.5)% at 2 °C to (41.9 ± 2.4)% at 45 °C. Literature does not clearly report the types and properties of native carbohydrase enzymes in soybeans, but our earlier study showed that *A. niger* carbohydrase activities increase with temperature to short-term optima between ~ 46 to 58 °C (Kabir and Ju 2023). Carbohydrate solubilization can result from both the non-enzymatic dissolution of originally soluble carbohydrates and the enzymatic hydrolysis of insoluble polysaccharides. The lack of temperature dependence observed here suggests that enzymatic hydrolysis of insoluble carbohydrates contributed minimally, and that the (46.3 ± 1.2)% solubilization corresponds roughly to the fraction of soluble carbohydrates naturally present in soybeans. In contrast, the strong temperature dependence of carbohydrate monomerization indicates that soybeans possess more active enzymes for hydrolyzing small soluble carbohydrates than for degrading structural polysaccharides.

Protein dissolution also showed a distinct temperature profile: a slow increase from 2 to 20 °C, a sharp rise at 20–25 °C, and a decline above 25 °C, reflecting the temperature sensitivity of soybean protease and its optimal functionality near 25 °C. This profile is consistent with the effect of temperature on soybean germination, which was also reported to be optimal around 25 °C (Szczerba et al. 2021; Begum et al. 2022). Protease plays a crucial role in seed germination, hydrolyzing storage proteins to provide nutrients for growth (Vorster et al. 2023). There are also reports on how heat stress affects the soybean production yield, including its disturbance of protein storage vacuole structure and membrane integrity (Krishnan et al. 2020). But there is no direct study on the effect of temperature on soybean protease. The protein dissolution results at different temperatures shown in Fig. 1 establishes an important first link between soybean protease activity and germination.

### Determination of limiting carbohydrase activities in SSF enzyme extracts for ESP

The analysis identified pectinase, polygalacturonase, and invertase as the three limiting activities that critically determine soybean particle breakdown during ESP. Pectinase and polygalacturonase were expected given the dominant role of pectin in soybean cell walls, whereas the importance of invertase was unexpected and points to an interdependence between structural and nonstructural carbohydrate hydrolysis.

Supporting data are summarized in Table 2. Volume reduction after 72 h of ESP varied widely (32–86%) depending on enzyme composition. ANOVA of all carbohydrases showed that the overall regression was statistically significant (n = 15, R^2^ = 0.975, F_9,5_ = 21.3, *p* = 0.002). Specifically, pectinase (*p* = 0.001), invertase (*p* = 0.021), and polygalacturonase (*p* = 0.041) significantly affected particle volume reduction, while other carbohydrases had no significant effects (*p* = 0.17–0.69). A second ANOVA restricted to these three enzymes yielded the regression equation:

$$\begin{aligned} & \% {\mathrm{particle}}{\mkern 1mu} {\mathrm{volume}}{\mkern 1mu} {\text{reduction = 0}}{\mathrm{.67}}\left( {{\mathrm{pectinase}}} \right) \\ & {\text{ + 0}}{\mathrm{.37}}\left( {{\mathrm{invertase}}} \right){\text{ + 1}}{\mathrm{.15(polygalacturonase)}} \\ \end{aligned}$$

where enzyme loadings are in U/g soybean. This model explained nearly all variations (n = 15, R^2^ = 0.995, F_3,12_ = 854.5, *p* < 0.001) and had very small correlation *p*-values (< 0.001, 0.001, and 0.009), reinforcing their critical roles in the enzymatic process.

These results align with soybean cotyledon structure. Pectin comprises ~ 50 to 70% of structural polysaccharides (Kohli and Singha 2024) and dominates the outer layer of cell wall (Zahir et al. 2020; Kohli and Singha 2024), making pectinase and its component polygalacturonase essential to initiate cell wall breakdown and expose hemicellulose and cellulose for further hydrolysis. The significance of invertase, however, was unexpected. Invertase hydrolyzes sucrose into glucose and fructose (Veana et al. 2018) and can cleave fructose from raffinose and stachyose (Kabir et al. 2025). Although there are no reports that these sugars directly inhibit structural polysaccharide hydrolysis, our finding suggests invertase indirectly facilitates ESP. A plausible hypothesis is that, by hydrolyzing sucrose and fructo-oligosaccharides associated with the cell wall matrix, invertase (1) disrupts their hydrogen-bonded associations and oligosaccharide-mediated cross-links that stabilize the wall, and (2) reduces their potential to hinder enzyme diffusion or water–polysaccharide interactions. These combined effects would help loosen the wall matrix and expose insoluble polysaccharides to enzyme attack—particularly by the limiting pectinase. These interpretations are consistent with reports that soluble oligosaccharides can stabilize cell wall polysaccharides (Van den Ende (Van 2013)) and that modification of wall components alters enzymatic accessibility (Refahi et al. (Refahi 2024)). Further study is needed to resolve these mechanisms.

Many systems in Table 2 showed > 60% volume reduction, under conditions of 0.5 M citrate buffer and high (5X) enzyme loading. The former increased protein release, as described later in Sect. “Effect of dilution medium”. The latter enabled more complete carbohydrate hydrolysis, giving high sugar concentrations in the hydrolysate. These conditions raised the specific gravity of hydrolysate, causing flotation of oil bodies, protein bodies, and some partially broken cells during centrifugation. Because volume reduction was measured from the height of bottom pellet, this flotation artifactually inflated values.

Taken together, these findings establish that ESP efficiency depends on both enzymes targeting structural polysaccharides (pectinase, polygalacturonase) and enzymes targeting soluble sugars (invertase). Boosting these activities during SSF could markedly improve enzymatic soybean biorefining.

### Effects of processing factors on protein loss

To minimize protein loss and avoid the need for separating proteins from sugars in the hydrolysate, it is highly desirable to limit protein dissolution during processing. The following sections present results from the evaluation of three processing factors aimed at achieving this objective.

#### Effect of protease in SSF enzyme extract on protein dissolution from SPI

**Fig. 2:**
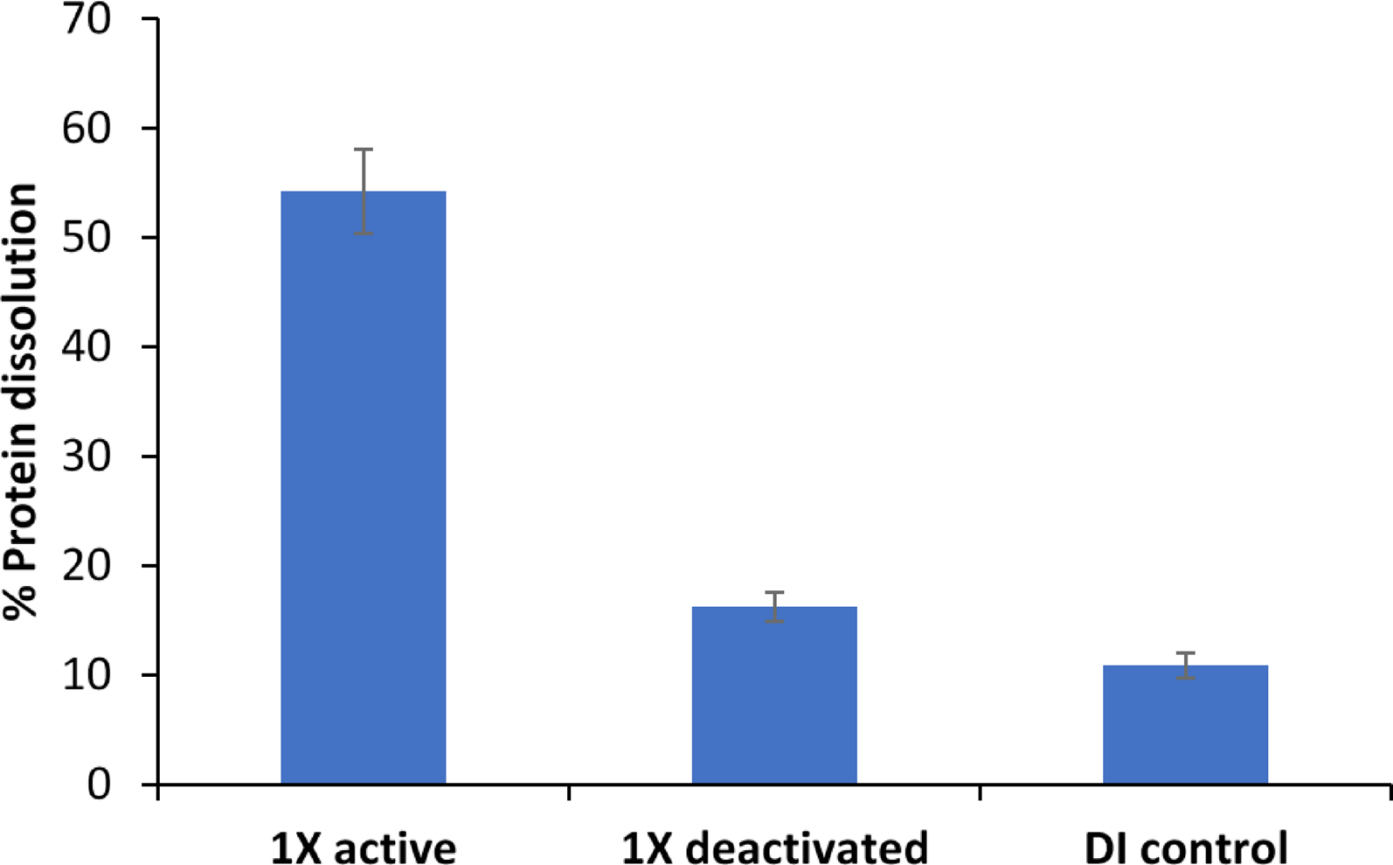
Protein dissolution of SPI by 1X SSF enzyme (Extract 1 in Table 2), compared with two controls: heat-deactivated enzyme extract and deionized water

Protein dissolution from SPI was significantly greater in the presence of the active SSF enzyme extract, reaching (54.2 ± 3.8)%, compared with (16.3 ± 1.3)% for the heat-deactivated extract and (10.8 ± 1.1)% for the deionized-water control (Fig. 2). These results demonstrate the critical contribution of protease activity in the SSF extract to protein loss during ESP.

The modest increase in protein dissolution with the deactivated extract relative to deionized water likely arose from non-enzymatic components of the SSF extract. Soluble organics, ions, and surface-active compounds present in the extract can destabilize protein structures or promote solubilization even in the absence of enzymatic activity (Stasiulewicz et al. 2022). Additionally, the extraction step introduced citrate buffer (final concentration 0.067 M), which may further contribute to protein solubilization.

In contrast, the 3.3-fold higher dissolution by the active enzyme extract underscores the predominant effect of protease. These findings highlight the importance of reducing protease levels in SSF enzyme production to preserve protein integrity during ESP.

#### Effect of processing time

**Fig. 3:**
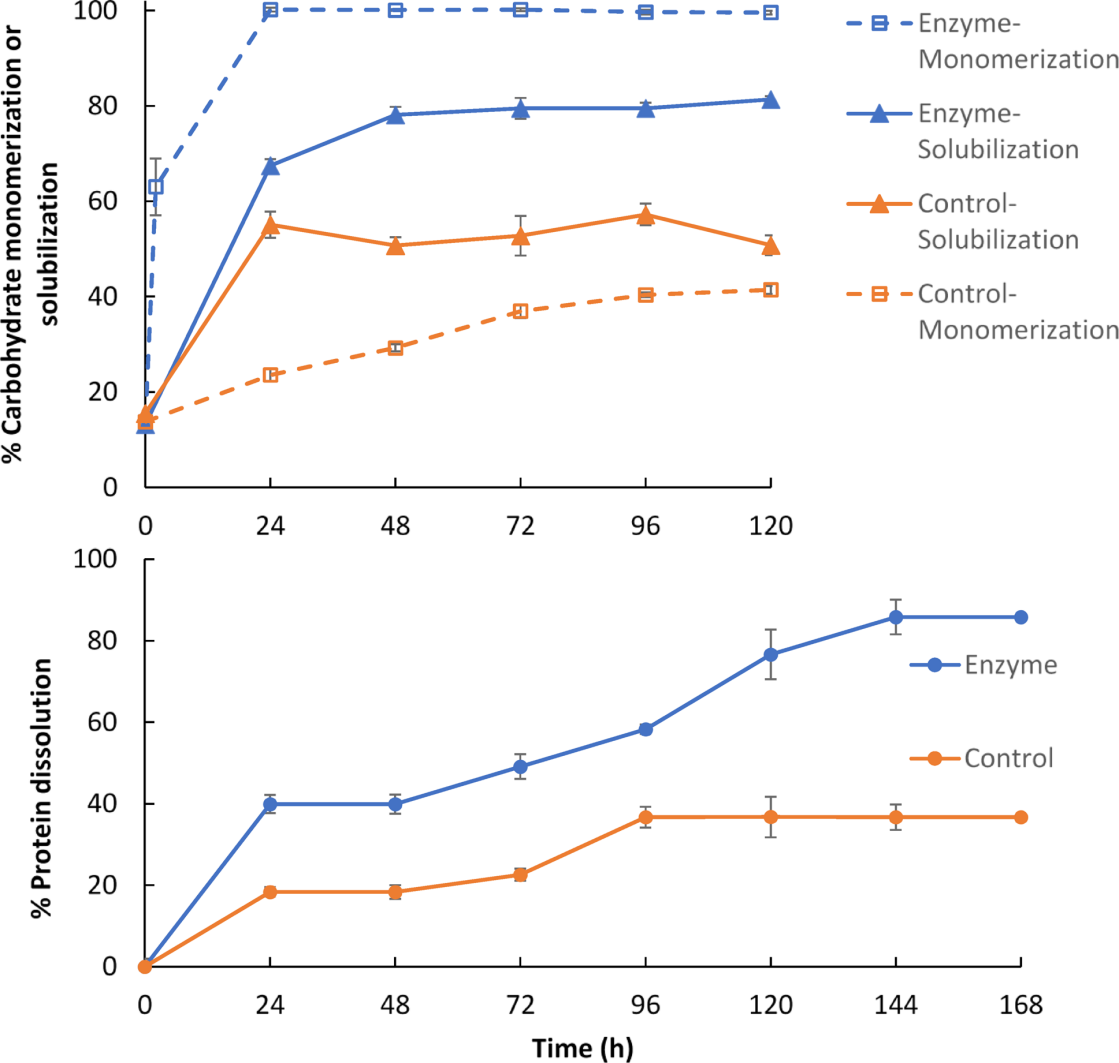
Profiles of carbohydrate solubilization and monomerization and protein dissolution in systems with and without 1X loading of an SSF enzyme extract

Processing time strongly influenced protein dissolution and carbohydrate conversion. With SSF enzyme extract, carbohydrate monomerization reached 100% within 24 h, whereas solubilization plateaued at ~ 80% after 48 h. In contrast, the control without enzyme reached only ~ 41% monomerization and ~ 53% solubilization. Protein dissolution showed a three-phase pattern: an initial rapid release, a stagnant phase, and then a gradual increase after 48 h that ultimately led to (85.8 ± 4.2)% protein loss with enzyme. These results indicate that extended ESP greatly increases protease-driven protein dissolution, underscoring the importance of limiting processing time to 1–2 days to reduce protein loss (Fig. 3).

Carbohydrate solubilization and protein dissolution can arise from two processes: (1) enzymatic hydrolysis of insoluble polymers into soluble oligomers and monomers, and (2) physical dissolution and diffusion of originally soluble or enzyme-solubilized compounds. By contrast, carbohydrate monomerization refers only to the enzymatic monomerization of already released soluble carbohydrates, unrelated to the release extent and mechanisms.

In the enzyme-free control, carbohydrate monomerization increased slowly from about 14% at the start to 41% after 120 h. This gradual rise indicates the presence of low levels of endogenous carbohydrases in soybeans that can monomerize soluble carbohydrates, consistent with the conclusion from Sect. “Carbohydrate release and monomerization and protein dissolution from soybean particles soaked in water” and Fig. 1. In the system containing SSF enzyme, by contrast, monomerization increased very rapidly, reaching 100% within 24 h. This shows that the SSF enzyme extract had high activities to monomerize all soluble and enzyme-solubilized carbohydrates released from the soybean particles.

Carbohydrate solubilization followed a different pattern. In the enzyme-free system, solubilization rose sharply only in the first 24 h and then plateaued at (53.3 ± 2.8)%, indicating that soybean cotyledons have negligible endogenous activities for hydrolyzing insoluble carbohydrates into soluble forms. Thus, the solubilization observed in the first day primarily reflected the release of pre-existing soluble carbohydrates. In the SSF enzyme system, solubilization also increased rapidly in the first 24 h, but then continued more slowly and plateaued at ~ 80% after 48 h—substantially higher than the ~ 53% seen in the enzyme-free control. Together with the results in Sect. “Determination of limiting carbohydrase activities in SSF enzyme extracts for ESP”, this finding points to limited pectinase (including polygalacturonase) activity in the SSF enzyme extract. Because pectin dominates soybean cotyledon cell walls, insufficient pectin hydrolysis would restrict access to cellulose and hemicellulose, thereby constraining overall carbohydrate solubilization.

Protein dissolution followed a three-stage pattern:

Initial phase (0–24 h): In the enzyme-free control, protein dissolution rose rapidly to (18.4 ± 0.9)%, likely reflecting the release of amino acids, small peptides, and proteins from the disrupted outer cell layers of soybean particles (caused by dehulling/cracking). The TKN method used here measures total nitrogen—including free amino acids and peptides, not just intact proteins—which helps explain this initial increase. In the SSF enzyme system, dissolution was substantially higher (39.9 ± 2.0)% at 24 h. This greater loss can be attributed both to non-enzymatic factors in the extract, which promote protein dissolution even from SPI (Fig. 2), and to protease activity hydrolyzing proteins in the disrupted outer layers.

Stagnant phase (24–48 h): Both the control and enzyme systems entered a plateau, suggesting depletion of readily soluble amino acids, peptides, and proteins, and a transition to slower release mechanisms.

Gradual increase (48–96 h in the control; extended to 144 h in the enzyme system): Protein dissolution resumed as amino acids, peptides, and proteins—including those generated by protease hydrolysis—diffused from deeper cell layers. In the control, this increase, driven by soybean’s endogenous protease, lasted until 96 h and reached (36.7 ± 5.0)% dissolution. The higher dissolution compared to the (17.3 ± 1.2)% in Fig. 1 reflects the use of 0.5 M citrate buffer here (vs. deionized water in Fig. 1), which promoted solubilization (discussed further in Sect. “Effect of dilution medium”). In the enzyme system, dissolution continued rising until 144 h, ultimately reaching (85.8 ± 4.2)%. This greater extent resulted from SSF protease acting on proteins newly exposed by enzymatic weakening and fracture of inner cell walls.

Together, these observations show that extended ESP substantially increases protein loss to the aqueous phase. By contrast, limiting processing to 1–2 days avoids the third phase dominated by protease hydrolysis and mass transfer, thereby preserving protein yield while still achieving high carbohydrate solubilization and complete monomerization.

#### Effect of dilution medium

**Fig. 4:**
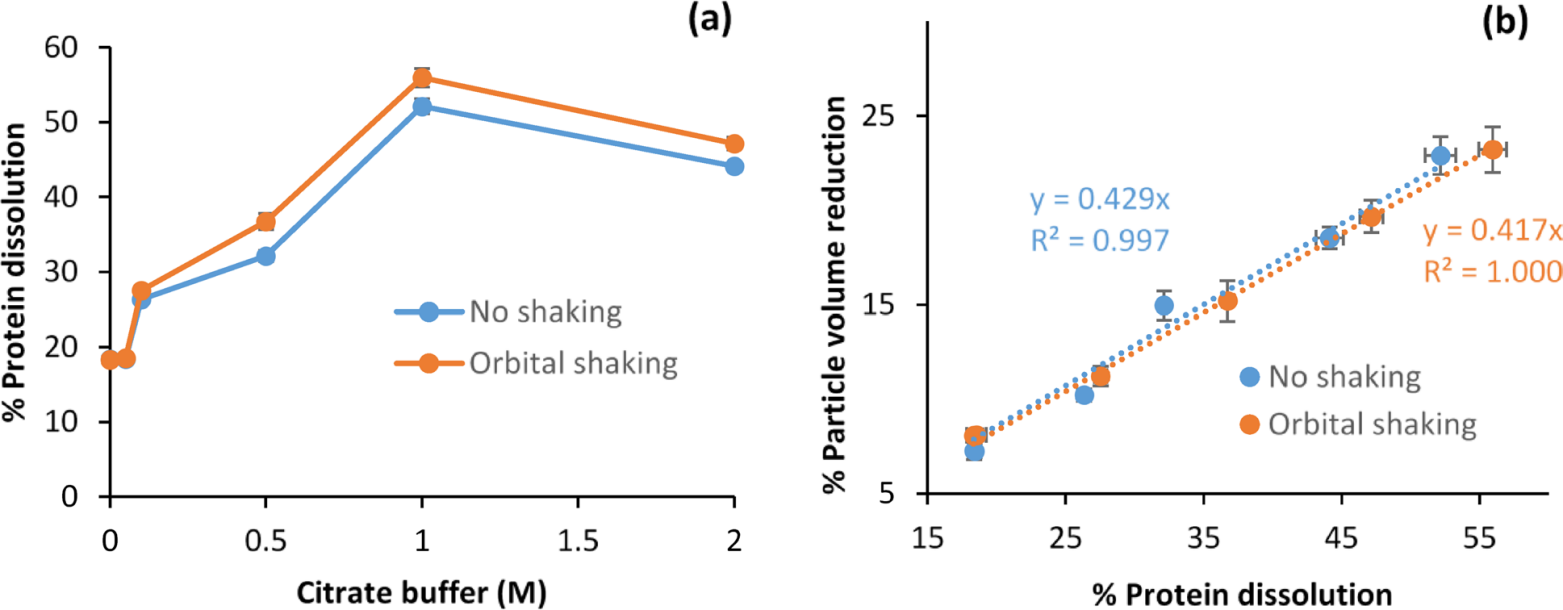
% Protein dissolution into pH 4.8 citrate buffers without SSF enzyme extract under no or 250 rpm orbital shaking: **a** effect of different citrate buffer concentrations and **b** effect on % particle volume reduction after 25 min of centrifugation at 4648 g. The systems had 1 g soybean particles soaked at 45 °C for 96 h in 10 mL buffer of 0 (deionized water), 0.05, 0.1, 0.5, 1, or 2 M concentration

Protein dissolution from soybean particles depended strongly on citrate buffer concentration, with negligible effect at ≤ 0.05 M but rising sharply at higher concentrations, whereas orbital shaking had only a minor influence (Fig. 4a). After 96 h at 45 °C, protein dissolution increased from 18.3% in deionized water to 52% and 56% (± 1%) in 1 M buffer without and with shaking, respectively, before declining slightly to 47% and 44% at 2 M. Shaking at 250 rpm had little impact (≤ 1%) when buffer concentration was ≤ 0.1 M but increased dissolution modestly (3.0–4.6%) at 0.5–2 M.

The enhanced protein dissolution in citrate buffer can be explained by a combination of general ionic strength effects and citrate’s specific multivalent interactions. At low-to-moderate ionic strength, citrate provides electrostatic screening that suppresses attractive patch–patch interactions and promotes protein solubility, consistent with the classical “salting-in” regime (Collins 2004). Beyond this, citrate’s trivalency enables selective binding to positively charged surface regions, altering local charge distribution and disrupting directional interactions that drive protein aggregation near the isoelectric point (Matsarskaia et al. 2020). For soybean proteins, aggregation near pI is known to involve both electrostatic patch pairing and hydrophobic exposure of the 7S and 11S subunits (Renkema et al. 2002; Nishinari et al. 2014). Citrate’s combined buffering, screening, and patch-specific effects provide a plausible mechanistic explanation for the observed increase in protein dissolution from ~ 18% in deionized water to > 50% in citrate buffer.

The changes in protein dissolution were closely associated with particle volume reduction. As shown in Fig. 4b, plotting % volume reduction against % protein dissolution gave nearly perfect linear correlations for both shaken and unshaken systems, with slopes of 0.417 (R^2^ = 1.000) and 0.429 (R^2^ = 0.997), respectively. Interestingly, these slopes are almost identical to the protein content of soybeans—reported as 43% in cotyledons (Medic et al. 2014) and measured here as (42.8 ± 1.0)% by TKN analysis.

Carbohydrate release was also influenced by citrate buffer concentration and by the buffer-to-soybean ratio. In another set of enzyme-free systems (10 mL, 45 °C, 250 rpm, 96 h), carbohydrate solubilization increased from (46.3 ± 2.0)% in deionized water to (60.2 ± 1.9)% in 1 M citrate buffer, with intermediate values of (48.1 ± 2.7)% and (57.2 ± 2.3)% at 0.1 and 0.5 M, respectively. Carbohydrate solubilization was also compared at two buffer volumes: 1 g soybean particles in 5 vs. 10 mL of 0.5 M citrate buffer. % solubilization was much lower at the smaller volume (25.6 ± 2.4% vs. 57.2 ± 2.3%), but the absolute sugar concentrations released (14.9 ± 2.0 vs. 16.6 ± 0.9 g/L) were statistically indistinguishable (*p* = 0.26). This indicates that carbohydrate release depends not only on buffer strength but also on aqueous-phase sugar concentration, which likely equilibrates with the concentration inside soybean particles. These findings are consistent with Long and Gibbons (2012), who reported 52–71% carbohydrate solubilization in 0.1 M citrate buffer under similar conditions (35 °C, 250 rpm, 96 h) but with a much larger buffer-to-soybean ratio (20 vs. 10 or 5 here). Their higher solubilization fits the same trend observed in this study, where increasing buffer-to-soybean ratios promoted greater % carbohydrate solubilization.

For practical ESP, the key implication is that using deionized water or very dilute citrate buffer (≤ 0.05 M) minimizes protein dissolution and allows maximal recovery of intact protein bodies. Although citrate buffers were originally applied to stabilize pH during ESP, subsequent experiments using deionized water as dilution medium showed that pH remained in a suitable range. Initial pH values were 4.3–5.1 (reflecting SSF enzyme extract), and after 96 h of processing, final pH rose only ~ 0.3 units (to 4.6–5.4). This demonstrates that strong buffering is unnecessary, and water alone is sufficient to maintain conditions for efficient ESP while preserving protein yield. In addition to simplifying operations, water-based processing also avoids the cost and waste streams associated with large quantities of buffer salts, making the approach both economically and environmentally advantageous. When considered together with the effects of processing time (Sect. “Effect of processing time”), these results show that ESP using water and limited duration (1–2 days) provides an effective compromise: protein yield is preserved while carbohydrate solubilization reaches near-maximum levels. This balance offers a practical strategy for guiding industrial applications where both protein recovery and carbohydrate monomerization are valued.

### Sonication-facilitated enzymatic soybean processing (ESP)

The results of this study demonstrate that pulsed probe sonication can markedly accelerate ESP while maintaining low protein dissolution, in contrast to orbital shaking or bath sonication that required much longer processing times. According to the findings from protein loss studies, sonication was evaluated for possibility of enhancing processing effectiveness and reducing the required processing time so as to minimize protein dissolution into the hydrolysate. Additionally, deionized water was used as the dilution medium. Two sonication setups were tested: bath sonication (0.15 W per mL bath volume, 10-min on/60-min off cycles, 96 h) and probe sonication (12 W per mL ESP mixture, 1-s on/23-s off cycles, 24 h). Systems processed for 96 h under orbital shaking were included for comparison.

**Fig. 5:**
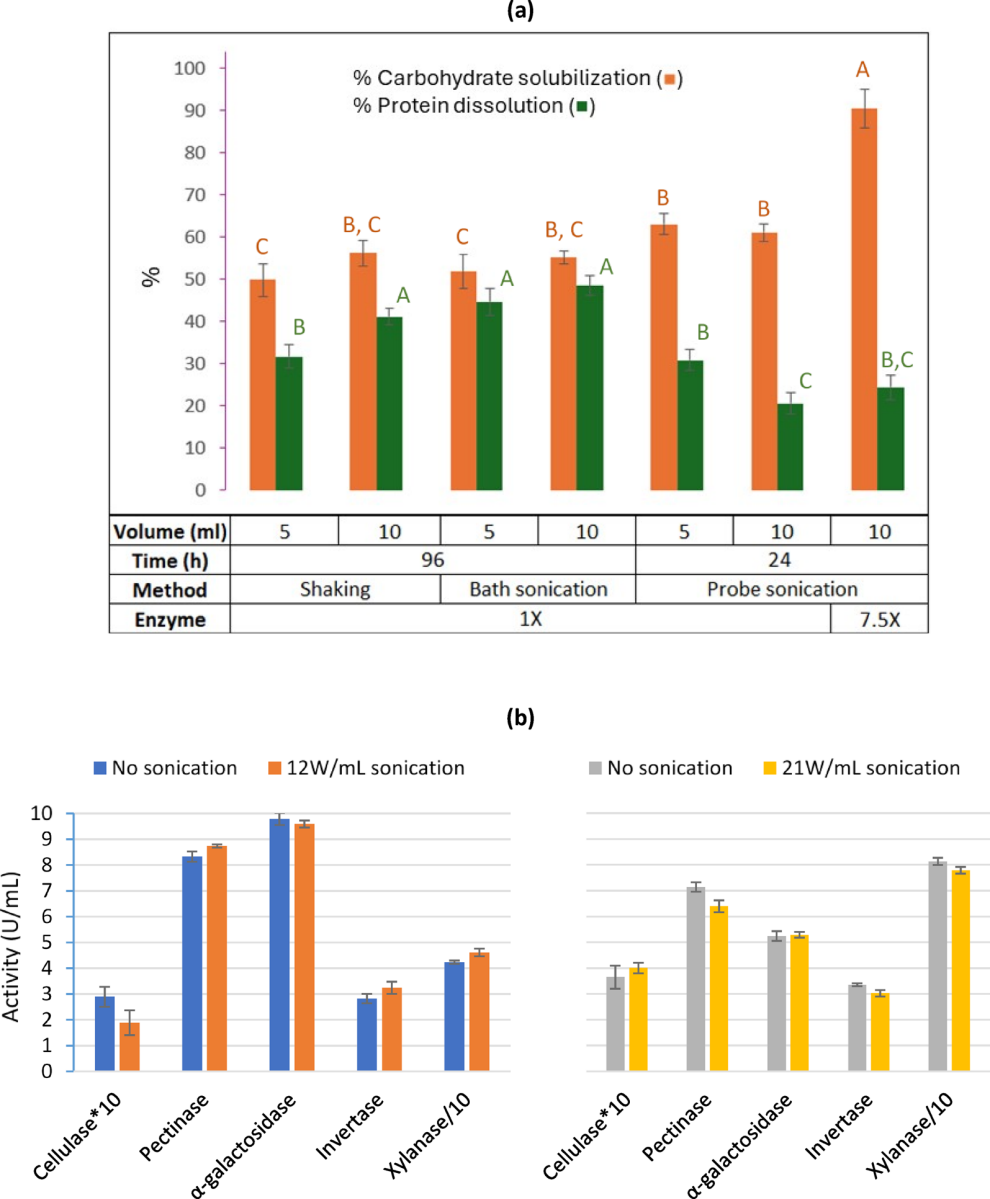
**a** % carbohydrate solubilization and % protein dissolution after 96 h of 250-rpm orbital shaking or low-intensity (0.15 W/mL) bath sonication, and after 24 h of higher-intensity (12 W/mL) probe sonication. Reactions were conducted in 5 or 10 mL volumes with either 1X or 7.5X loading of enzyme extract 1 (Table 2). Statistical groupings based on Tukey’s honestly significant difference (HSD) test are indicated by letter labels. Means sharing the same letter are not significantly different at *p* < 0.05. **b**. Carbohydrase activities were not significantly affected by pulsed probe sonication (1 s on / 23 s off for 24 h) at 12 and 21 W/mL intensity (during “on” phase)

Results of % carbohydrate solubilization and % protein dissolution are shown in Fig. 5a. Results for % carbohydrate monomerization—which refers to the percentage of soluble carbohydrates in the hydrolysate present as monomeric sugars—are not shown because monomerization reached 100% in all systems. This confirms that the SSF enzyme extract provided excess activity for converting soluble oligosaccharides to monomers. However, % carbohydrate solubilization—which refers to the percentage of total soybean carbohydrate made soluble (by dissolution or enzymatic hydrolysis)—differed considerably. Among 1X enzyme systems, orbital shaking and bath sonication gave statistically similar carbohydrate solubilization after 96 h: (49.8 ± 4.0)% vs. (51.8 ± 4.0)% for 5 mL (*p* = 0.63, both Tukey group C in Fig. 5a) and (56.2 ± 3.0)% vs. (55.1 ± 1.6)% for 10 mL (*p* = 0.71, both in overlapping groups B/C). In contrast, probe-sonicated systems (5 mL, 1X enzyme, 24 h) achieved significantly higher carbohydrate solubilization (Tukey group B), about 24% more than the corresponding shaken or bath-sonicated systems, despite requiring only one quarter of the processing time. Reaction volume (5 vs. 10 mL) did not significantly affect carbohydrate solubilization in enzyme-treated systems.

Protein dissolution showed a different pattern. Under orbital shaking, protein dissolution increased with reaction volume, from (31.7 ± 2.8)% in 5 mL (Tukey group B) to (41.1 ± 2.0)% in 10 mL (Tukey group A). Bath sonication yielded similar dissolution levels in both volumes (Tukey group A), and in the 5 mL systems, it produced somewhat greater dissolution than orbital shaking. More importantly, under the probe sonication with a much shorter processing time (24 vs. 96 h), protein dissolution remained similar to orbital shaking in the 5 mL system (Tukey group B) and was much lower in the 10 mL system ((20.5 ± 2.6)%, Tukey group C). Thus, probe sonication enhanced carbohydrate solubilization without worsening protein loss. Under probe sonication, increasing the enzyme loading to 7.5X gave (90.4 ± 4.6)% carbohydrate solubilization within 24 h, while maintaining low protein dissolution at (24.4 ± 2.9)%. Such extensive carbohydrate solubilization was sufficient to rupture essentially all soybean cell walls and release protein bodies and oil bodies for separate collection, as described in the next section.

To evaluate whether probe sonication would compromise enzyme stability, activities of two enzyme extracts were measured before and after 24 h of pulsed treatment at 12 and 21 W/mL (during the “on” phase). As shown in Fig. 5b, no substantial loss of carbohydrase activity was observed. The pulsed regime (1 s on/23 s off) likely reduced thermal and mechanical stresses, allowing enzymes to remain active while benefiting from cavitation and microstreaming that enhanced mass transfer and substrate accessibility.

Taken together, these results indicate that probe sonication, when applied in a pulsed mode, provides a practical means of accelerating ESP. Rapid carbohydrate solubilization and complete monomerization can be achieved within 24 h, while protein dissolution remains controlled. The combination of optimized enzyme loading and probe sonication thus enables efficient breakdown of cell walls and release of internal components in a fraction of the time required under orbital shaking.

In this study, pulsed sonication (1 s on/23 s off for 24 h) ensured that almost the entire small sample volume was in the active sonication zone during each pulse, yielding an effective cumulative sonication time of ~ 1 h. Pulsing also minimized heat buildup, which is important for avoiding enzyme deactivation during long processing times. At larger scales, pulsing may be unnecessary provided that mixing or recirculation moves different portions of the bulk sample through the active zone. For instance, a probe could be placed in a mixed tank where gentle agitation continuously exchanges material into the sonication field, or a side-stream could be circulated through a sonication chamber at a controlled flow rate. For pilot-scale design, a reasonable starting point is to target an average cumulative residence time of ~ 1 h in the active zone, with optimization of mixing intensity or circulation speed as needed. In such systems, energy efficiency will depend on minimizing redundant exposure and controlling local heating, both of which can be managed by balancing mixing and flow conditions. While we have not conducted scale-up studies, this framework provides an initial basis for applying pulsed-sonication parameters to continuous processing.

### Product separation

**Fig. 6:**
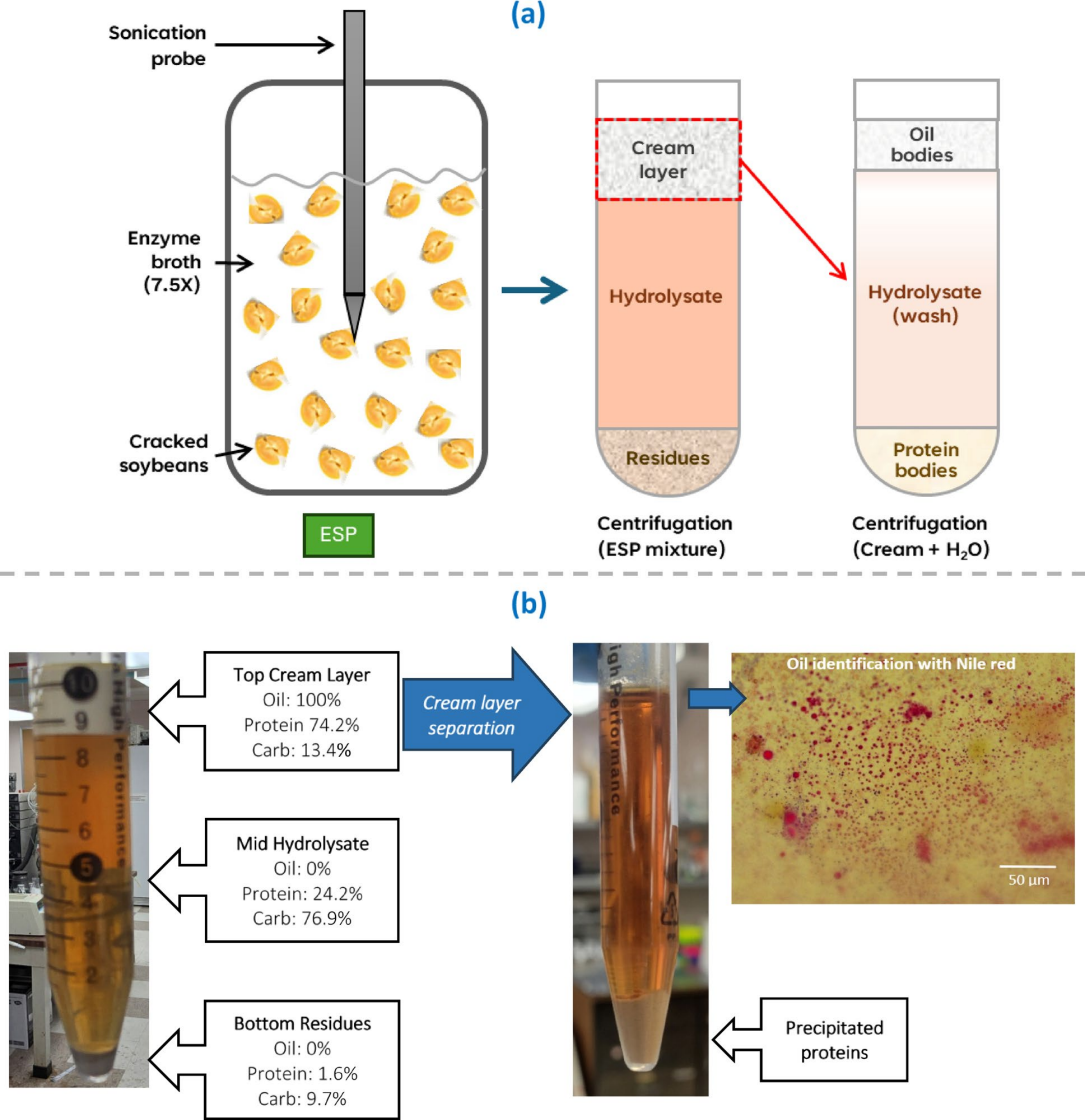
**a** Schematic overview of ESP and centrifugation steps leading to product fractionation. **b** Product separation after 24 h of ESP with pulsed probe sonication and 7.5X enzyme loading. Following an initial 25-min centrifugation at 4648 × g, three distinct layers formed (left). The cream layer was collected, diluted with deionized water, and subjected to a second centrifugation (4648 × g, 25 min) to precipitate proteins and separate floating oil bodies (right)

A major advantage of the ESP approach is that, unlike conventional soy processing where product separation is complex and energy-intensive (Loman and Ju 2017), centrifugation was sufficient to recover distinct product fractions. After 24 h of ESP with pulsed probe sonication and 7.5X enzyme loading (described in Sect. “Sonication-facilitated enzymatic soybean processing (ESP)”), the mixture separated into three layers: a cream-like top layer, a large aqueous hydrolysate in the middle, and a small bottom precipitate (Fig. 6). The top layer contained essentially all of the soybean oil, 74.2% of (all) protein, and 13.5% of carbohydrate. The hydrolysate contained 24.2% of protein and 76.8% of carbohydrate, while the bottom precipitate carried only 1.4% of protein and 9.7% of carbohydrate. Because these measurements were made with wet samples, some soluble proteins and carbohydrates were entrapped within the top and bottom layers. However, given the much higher ratio of soluble carbohydrate to soluble protein in the hydrolysate, most of the protein in the top layer was insoluble protein bodies, whereas most of the carbohydrate in the bottom layer was residual non-solubilized polysaccharides.

The flotation of protein bodies was due to the increased density of the hydrolysate at high enzyme loading. In 1X enzyme systems, where hydrolysate density was lower, protein bodies mainly precipitated into the bottom layer. This interpretation was confirmed by re-suspending the top layer from the first centrifugation in deionized water and centrifuging again. The reduced hydrolysate density caused protein bodies to precipitate, while oil bodies floated to the surface. This second step also released entrapped soluble proteins and carbohydrates into the new hydrolysate, improving recovery.

The identity of the floated oil bodies was verified by Nile Red staining. Samples from the oil-rich top layer were split: one was examined at native pH and the other at pH 12 (adjusted using NaOH). Under native pH, oil bodies remained small and not microscopically visible at 400 × magnification, but after alkaline disruption they coalesced into larger droplets that stained brightly, confirming their presence (Fig. 6). These observations demonstrate that ESP liberates intact oil bodies, which can be recovered without solvent extraction or chemical modification.

Overall, ESP hydrolyzes most cell-wall polysaccharides into soluble monosaccharides, releasing storage proteins and oil bodies from cotyledon cells. Following centrifugation, insoluble proteins and oil bodies can be collected in the top layer, provided the hydrolysate density is sufficient; otherwise, proteins precipitate with residual waste in the bottom layer. A second centrifugation after dilution with deionized water efficiently separates oil bodies (floating) from proteins (precipitating), while simultaneously releasing entrapped solubles. As illustrated in Fig. 6, this two-step centrifugation enables recovery of ~ 70% of protein and essentially 100% of oil bodies as nearly pure product streams, along with a hydrolysate containing the remaining soluble protein (> 25%) and ~ 90% of carbohydrate, entirely as value-added monosaccharides.

Table 3 compares this study with eight recent reports (2021–2025) on aqueous enzymatic processing for oil and/or protein recovery, with and without sonication. Most of these previous studies targeted bulk oil extraction rather than intact oil bodies. One study focused solely on protein recovery from quinoa (Yang et al. 2024), and only one aimed at simultaneous oil and protein recovery (almond flour; Dias et al. 2022). Compared to these works, the present study demonstrates competitive or superior recovery of both intact oil bodies and functional protein.

**Table 3:** Comparative Studies on Aqueous Enzymatic Processing (AEP), without and with Sonication, for Oil/Protein Recovery

Feedstock	Enzyme(s)/Process	Sonication	Objective	Product Quality/Integrity	Oil Yield (%)	Protein Yield (%)	References
Argan kernels	Viscozyme (carbohydrases), pectinase, or cellulase	No	Compare conventional vs enzyme-assisted extraction	Bulk oil; quality preserved; low oxidation	66.4 (Viscozyme); 52 (pectinase); 40 (cellulase)	–	(Mechqoq et al. 2021)
Almond flour (full-fat)	Neutral protease; AEP + emulsion destabilization	No	Simultaneous oil & protein extraction	Oil composition unchanged; protein partially hydrolyzed	67 (AEP); 93 after demulsification	77	(Dias et al. 2022)
Olives (cv. Coratina)	Pectinase, PME & PG; talc	No	Industrial oil extraction; improve yield and phenolic content	Bulk oil; phenolic increased (12–16%); volatiles unchanged	86.9 (control), 89.7 (enz), 89.2 (talc), 92.5 (enz + talc)	–	(Tamborrino et al. 2023)
Macauba pulp	Pectinase	No	AEP vs. mechanical pressing & solvent extraction	Bulk oil; low acidity; bioactives preserved	88.6 (AEP)	–	(Sorita et al. 2024)
*Thevetia peruviana* seeds	Alcalase, Viscozyme L, cellulase	No	Oil for biodiesel/industrial use	Bulk oil; improved oxidative stability vs hexane	Up to 78.2	–	(Domínguez-Pérez et al. 2025)
Quinoa	Alkaline protease; sonication-assisted AEP	Yes	Enhance protein yield & functionality	Improved protein solubility, emulsifying, foaming	–	26.7 (max)	(Yang et al. 2024)
Hemp seed	Pectinase & hemicellulase; ultrasound-ethanol pretreatment	Yes	Maximize oil yield, reduce psychoactives	Bulk oil	Up to 88.4	–	(Zhang et al. 2024a)
High-oleic & normal peanuts	Viscozyme L; sonication-assisted AEP	Yes	Regulate interfacial protein on oil bodies	Intact OBs; improved interfacial protein (88 → 92%)	Up to ~ 87 OB	–	(Wang et al. 2025)
Soybean particles	Carbohydrase cocktail from *A. niger* SSF; sonication-assisted AEP	Yes	Sustainable soy processing; recover OBs, protein & sugars	Intact OBs, undenatured protein, ~ 90% sugars	Up to ~ 100 OB	~ 70 insoluble (pH 4.8); ~ 25 aqueous	This study

PME, pectinmethylesterase; PG, polygalacturonase

### Industrial relevance and potential value of recovered fractions

While a rigorous techno-economic or life-cycle assessment is premature at this stage, some indicative comparisons highlight the potential industrial relevance of ESP. Conventional hexane extraction requires make-up solvent on the order of 0.5–0.8 kg hexane per tonne of soybeans processed, representing a recurring material input as well as an energy demand for solvent recovery (Loman and Ju 2017). More importantly, by eliminating hexane altogether, ESP not only avoids solvent purchase but also removes volatile organic emissions and the need for solvent-handling infrastructure and desolventizing/distillation steam, thereby reducing both capital and operating burdens.

The recovered product fractions also have potential to command higher values than their conventional counterparts. Commodity soy protein concentrates (SPC) are sold in the $0.9–5.2/kg range, while SPI are typically in the $2.0–15.0/kg range depending on purity and market (Tridge 2025). By contrast, undenatured “native” proteins are niche but substantially more valuable; for example, undenatured/native whey isolates are retailed at $30–50/kg (Nutristat 2025), reflecting a premium for preserved functionality. No equivalent market data are publicly available for undenatured soy protein, but a similar premium appears plausible.

Likewise, intact oil bodies are not yet traded as bulk commodities, but they are already incorporated into cosmetic and personal-care formulations (e.g., Sharon Personal Care using Botaneco oleosome ingredients), where they are valued as natural emulsifiers and delivery systems. This is in sharp contrast to commodity crude soybean oil, which typically sells at $1.0–1.2/kg (FAO 2024). Finally, ESP converts oligosaccharides into fermentable monosaccharides, avoiding low-value soybean molasses and instead producing a hydrolysate directly usable as fermentation feedstock (Loman et al. 2018a).

These illustrative comparisons underscore that ESP enables recovery of fractions with differentiated properties and potentially higher unit values than those obtained by conventional processing, even if exact market prices are not yet established.

## Conclusions

This study establishes a novel enzymatic soybean processing (ESP) strategy that enables the separate recovery of oil bodies, native proteins, and monomerized carbohydrates without thermal or solvent-based denaturation. Effective cell wall disruption was shown to depend on multiple carbohydrase activities, with pectinase, polygalacturonase, and invertase identified as limiting in *A. niger* SSF enzyme extracts. Protein preservation was achieved by minimizing protease activity, using shorter processing times, and replacing buffer with deionized water as an eco-friendly alternative. For the first time, pulsed sonication was integrated with ESP, accelerating carbohydrate solubilization and reducing processing time from days to hours while maintaining protein integrity. Overall, this integrated approach demonstrates a gentle, sustainable alternative to conventional soybean processing and provides a pathway consistent with circular bioeconomy models.

## Supplementary Information

Below is the link to the electronic supplementary material.

Supplementary Material 1

## Data Availability

The datasets used and/or analyzed during the current study are available from the corresponding author on reasonable request.
