# Supplementary material for: Integrated enzymatic and sonication strategy for sustainable soybean processing: from cell wall deconstruction to product separation
Source: Bioresour Bioprocess. 2026 Jan 27;13(1):6. doi: 10.1186/s40643-025-00991-5 (PMC12835480; doi:10.1186/s40643-025-00991-5)
Supplement: Supplementary file 1 — Supplementary Material 1 [file 40643_2025_991_MOESM1_ESM.docx]

Supplementary Materials

1. Protein analysis by Total Kjeldahl Nitrogen (TKN) method

For measuring protein content of an enzyme extract or hydrolysate, a 1 mL sample was placed in a 250 mL volumetric flask and digested with 10 mL of a reagent composed of 146.45 g/L K_2_SO_4_, 16.36 g/L CuSO_4_·5H_2_O, and 4.18 g/L selenium powders in concentrated H_2_SO_4_. The digestion occurred at 450°C until the mixture turned clear/transparent. The digested sample was then diluted to 20 mL with deionized water. From this, 2 mL was transferred to a distillation unit and then added with 10 N NaOH till neutral pH, with phenolphthalein as the titration indicator. The neutralized mixture was next distilled for 5 min to release gaseous NH_3_, which was absorbed in 20 mL of 0.1 N H_3_BO_3_. The resulting solution was titrated with 0.1358 N H_2_SO_4_ using a 1:1 methyl red and methylene blue indicator. The N content in the sample was calculated using the following equation:

N content (g) = $\frac{V \times14.007 \times N}{1000}$,

where V = volume (mL) of H_2_SO_4_ used to titrate the H_3_BO_3_ from sample – volume (mL) of H_2_SO_4_ used to titrate the H_3_BO_3_ of N-free control (deionized water), and N = 0.1358, the normality of H_2_SO_4_ used for H_3_BO_3_ titration. To measure N content in solid samples such as soybean particles (before or after processing) or SPI, an accurately weighed solid sample (<0.5 g) was used in place of the 1 mL liquid sample. To determine the % protein dissolution during soybean processing, the N content of the aqueous hydrolysate, after subtracting the background N contributed by the enzyme extract, was compared with the N content in the original soybean particles or SPI.

2. Analysis of enzyme activities

α-Galactosidase was assayed using the method of Kumar et al. (Kumar et al. 2012), modified by Li et al. (Li et al. 2017). The substrate solution was 0.333 g/L p-nitrophenyl-α-D-galactopyranoside (CAS #7493-95-0, Sigma Aldrich) in 0.1 M sodium citrate buffer (pH 4.8). For the assay, 900 μL of substrate was mixed with or, for control, without 100 μL of the sample and incubated at 50°C for 10 min. Then, 2 mL of 0.5 M sodium carbonate (pH 9.8) was added to stop the reaction. The control was added with 100 μL of the sample and used as the reference in subsequent absorbance measurement at 405 nm. The α-galactosidase activity was calculated using a calibration curve developed with pure *p*-nitrophenol standards.

Invertase was assayed using a modified method of Uma et al. (Uma et al. 2010), as adapted by Li et al. (Li et al. 2017), with 5 g/L sucrose (in 0.1 M pH 4.8 citrate buffer) as substrate. The preparation and incubation steps were like those for the α-galactosidase assay except for the longer incubation time of 20 min. After incubation, 3 mL of a DNS solution (10 g/L 3,5-dinitrosalicylic acid, 16 g/L NaOH, and 300 g/L sodium potassium tartrate) was added to terminate the reaction, and 100 μL of the sample was added to the control. The mixtures were heated in boiling water for 10 min, diluted to 25 mL with deionized water, and then measured for absorbance at 540 nm. Calibration with glucose standards was developed and used to determine the enzyme activity.

Pectinase, polygalacturonase, cellulase, and xylanase activities were determined using the methods developed by Li et al. (Li et al. 2017), Ghose (Ghose 1987), and Bailey et al. (Bailey et al. 1992), respectively, with procedures like the invertase assay but differing in substrates, incubation times, and calibration standards. For pectinase and polygalacturonase, the substrate was 5 g/L citrus pectin (P9135, galacturonic acid ≥74%, Sigma-Aldrich) or polygalacturonic acid (CAS #25990-10-7, Thermo Fischer, Waltham, MA) in 0.1 M citrate buffer (pH 4.8), with 30-min incubation and galacturonic acid (CAS #91510-62-2, Thermo Fischer) as the calibration standard. For cellulase, a rolled strip of Whatman No. 1 filter paper (6 × 1 cm) in 1.4 mL of 0.05 M citrate buffer (pH 4.8) served as the substrate, with 60-min incubation and glucose-based calibration. For xylanase, the substrate was 10 g/L beechwood xylan (CAS #9014-63-5, Avantor Sciences, Radnor, PA) in 0.05 M citrate buffer (pH 5.3), with 5-min incubation and xylose for calibration.

Assays for β-glucosidase (cellobiase), endoglucanase, and exoglucanase followed the methods reported by Ju and Afolabi (Ju and Afolabi 1999), modified from Wald et al. (Wald et al. 1984) and Berghem and Petterson (Berghem and Pettersson 1973; Gunjikar et al. 2001). For β-glucosidase assay, three test tubes were prepared: the first, a cellobiose (CAS #528-50-7, Thermo Fischer) blank, contained 1.0 mL each of 15 mM cellobiose solution, citrate buffer (pH 4.8), and water; the second, a sample blank, contained 1.0 mL of the sample and 2.0 mL of water; and the third, the test sample, contained 1.0 mL each of the cellobiose solution, buffer, and enzyme sample. All tubes were mixed, tightly sealed, incubated at 50°C for 30 min, added with 3 mL DNS reagent, and then measured for absorbance at 540 nm. The absorbance of the test sample, corrected by subtracting the values for the two blanks, was used to determine the glucose concentration released by the enzyme and subsequently converted to the β-glucosidase activity. For endoglucanase assay, the substrate solution was 1% carboxymethylcellulose (CMC, CAS #9004-32-4, Sigma-Aldrich) in 0.05 M sodium acetate buffer at pH 5. One mL of the substrate was added with 0.28 mL of the enzyme sample, incubated at 50°C for 30 min, added with 3 mL DNS reagent, and measured for absorbance at 540 nm. Accordingly, the concentration of reducing sugar released was determined using glucose as standard, and the endoglucanase activity was calculated. The exoglucanase assay was similar. One mL of the enzyme sample was added with 1 mL of a 2% Avicel (Product No. 11365, Sigma-Aldrich) suspension in 0.05 M sodium acetate buffer (pH 5), incubated at 40°C for 30 min, added with 3 mL DNS reagent, measured for absorbance at 540 nm, and determined for concentration of released reducing sugar and then exoglucanase activity.

Protease activity was measured using the Pierce Fluorescent Protease Assay Kit (Thermo Scientific, Catalog No. 23266), as described by Li et al. (Li et al. 2017). The assay employed FTC-casein (κ-casein labeled with fluorescein isothiocyanate to form a fluorescein thiocarbamoyl [FTC] derivative) as the protein substrate and TPCK-treated trypsin as the reference protease. For the assay, a substrate solution was prepared by mixing 20 μL of freshly prepared 5 g/L FTC-casein with 10 mL of 0.05 M sodium citrate buffer (pH 4.8). Fifty μL of this substrate solution and 20 μL of an enzyme sample or TPCK trypsin standard were combined in a 96-well black plate (Greiner Bio One, Catalog Number 655076), mixed, and allowed to react at room temperature for 20 min. To terminate the reaction and raise the pH for fluorescence measurement, 200 μL of 1 M Tris–HCl (pH 9.0) was added. Fluorescence was measured using a multimode microplate reader (Infinite 200 PRO, TECAN) at an excitation wavelength of 485 nm and an emission wavelength of 538 nm. A calibration curve was established using TPCK trypsin standards at concentrations ranging from 0 to 50 mg/L. The fluorescence change from the enzyme samples was converted to an equivalent TPCK trypsin concentration and subsequently expressed in BAEE U/mL by multiplying by a factor of 16,273.
